# Extracellular vesicles derived from Krüppel-Like Factor 2-overexpressing endothelial cells attenuate myocardial ischemia-reperfusion injury by preventing Ly6C^high^ monocyte recruitment

**DOI:** 10.7150/thno.45459

**Published:** 2020-09-18

**Authors:** Shuaihua Qiao, Wenfeng Zhang, Yong Yin, Zhonghai Wei, Fu Chen, Jinxuan Zhao, Xuan Sun, Dan Mu, Jun Xie, Biao Xu

**Affiliations:** 1 Department of Cardiology, Nanjing Drum Tower Hospital, State Key Laboratory of Pharmaceutical Biotechnology, Medical School of Nanjing University, Nanjing, China.; 2 Department of Radiology, Nanjing Drum Tower Hospital, State Key Laboratory of Pharmaceutical Biotechnology, Medical School of Nanjing University, Nanjing, China.; 3 Department of Cardiology, Nanjing Drum Tower Hospital Clinical College of Nanjing Medical University, Nanjing, China.

**Keywords:** Krüppel-Like Factor 2, extracellular vesicles, myocardial ischemia/reperfusion injury

## Abstract

**Background:** The ischemia/reperfusion (I/R) process in patients with ST-segment elevation myocardial infarction (STEMI) triggers an immune response, resulting in myocyte death. Krüppel-Like Factor 2 (KLF2), which is highly expressed in endothelial cells (ECs) under laminar flow, exerts anti-inflammatory effects. In this study, we explored the role of small extracellular vesicles (EVs) from KLF2-overexpressing ECs (KLF2-EVs) in the immunomodulation and its implications in myocardial I/R injury.

**Methods and Results:** The small EVs were isolated from KLF2-overexpressing ECs' supernatant using gradient centrifugation. Mice were subjected to 45 min of ischemia followed by reperfusion, and KLF2-EVs were administrated through intravenous injection. KLF2-EVs ameliorated I/R injury and alleviated inflammation level in the serum and heart. We employed the macrophage depletion model and splenectomy and showed that Ly6C^high^ monocyte recruitment from bone marrow was the main target of KLF2-EVs. miRNA-sequencing of KLF2-EVs and bioinformatics analysis implicated miRNA-24-3p (miR-24-3p) as a potent candidate mediator of monocyte recruitment and CCR2 as a downstream target. miR-24-3p mimic inhibited the migration of Ly6C^high^ monocytes, and miR-24-3p antagomir reversed the effect of KLF2-EVs in myocardial I/R.

**Conclusion:** Our data demonstrated that KLF2-EVs attenuated myocardial I/R injury in mice via shuttling miR-24-3p that restrained the Ly6C^high^ monocyte recruitment. Thus, KLF2-EVs could be a potential therapeutic agent for myocardial I/R injury.

## Introduction

Ischemic heart disease is the leading cause of death and disability worldwide 1. Currently, reperfusion therapy with the primary percutaneous coronary intervention (PCI) and fibrinolytic drugs for ST-segment elevation myocardial infarction (STEMI) patients could restore blood flow to ischemic myocardium and reduce infarct size. Nevertheless, reperfusion induces an immune response and myocardial damage, known as reperfusion injury. The ischemia/reperfusion (I/R) process triggers the innate response, including neutrophils and monocytes, which play an important role in both myocardial injury and healing 2. Accordingly, two distinct waves of monocyte recruitment have been identified in healing myocardial infarct 3. Early recruitment of pro-inflammatory Ly6C^high^ monocytes is mediated by the angiotensin type 1 receptor (Atgr1) interacted with angiotensin II (Ang II) from the spleen 4 and then is enabled through the activation of the monocyte chemoattractant protein-1 (MCP-1)/C-C chemokine receptor type 2 (CCR2) axis from bone marrow 5. At a later stage, anti-inflammatory Ly6C^low^ monocytes are accumulated in infarct myocardium by fractalkine/CX3C chemokine receptor 1 (CX3CR1) axis or are transduced from Ly6C^high^ monocytes, participating in the resolution of the post-infarction inflammatory response 6. Ly6C^high^ monocyte homing is necessary for necrotic cell clearance and tissue repair. However, under the I/R status, these pro-inflammatory monocytes are excessive and thereby constitute a major cause of subsequent myocyte death and myocardium dysfunction 7, 8. Thus, an optimal, but not excessive level of Ly6C^high^ monocyte activation would be more appropriate for ischemic myocardium repair and better prognosis.

Endothelial cells (ECs) are considered as major functional coordinators in the vascular homeostasis, especially in anti-inflammatory and anti-thrombotic processes 9. ECs regulate the inflammatory cells in peripheral blood via a paracrine mechanism in physiological status 10. Under pathological stress, ECs divert to a pro-inflammatory phenotype and participate in inflammatory cell activation and homing 11. Krüppel-Like Factor 2 (KLF2) is identified as a key “molecular switch” for the ECs to maintain anti-inflammatory phenotype and is activated by laminar flow through a mechanosensory complex 12. Previous studies showed that KLF2-transduced ECs could exert anti-inflammatory effect and mediate monocyte/macrophage (Mo/Mø) polarization in atherosclerosis 13, indicating that KLF2-modified ECs could be used for the treatment of inflammatory diseases.

To date, there are no effective anti-inflammation therapies to suppress myocardial I/R injury. Extracellular vesicles (EVs), which are centrally involved in intercellular communication 14, are thought to be potential biological mediators to exert therapeutic effects on diseases. In contrast to cell therapy, small-sized EVs have many benefits, including biocompatibility, non-toxicity, immunological inertness, escape from phagocytosis, and the inherent capacity to pass through biological barriers 15. Recently, EVs derived from KLF2-transduced ECs were reported to reduce atherosclerotic lesion formation in the aorta of ApoE^-/-^ mice 16. Another study has shown that EVs from endothelial progenitor cells improved outcomes of the lipopolysaccharide-induced acute lung injury 17. Hence, whether EVs from KLF2-transduced ECs could be used as a potential therapeutic agent to attenuate inflammation in myocardial I/R injury needs to be explored.

In this study, we isolated EVs from KLF2-overexpressing ECs (KLF2-EVs), which were intravenously injected into mice immediately after the myocardial I/R injury. We found that KLF2-EVs could prevent Ly6C^high^ monocyte recruitment to relieve inflammatory cascade and decrease myocardial infarct size, mediated via miRNA-24-3p (miR-24-3p) by inhibiting CCR2 expression.

## Methods

Additional materials and methods are shown in the Supplementary Methods.

### EVs isolation and characterization

The human umbilical vein endothelial cells (HUVECs) (Science Cell, 19196) were cultured in endothelial cells (ECs) culture medium (Science Cell, 1001) supplemented with 10% fetal bovine serum (FBS, Science Cell, 0025), 1% endothelial cell growth supplement (Science Cell, 1052) and 1% Penicillin/Streptomycin (Science Cell, 0503). The mouse coronary endothelial cells (MCAECs) were isolated from mouse coronary endothelium according to the protocol and video 18. When the ECs reached 60-70% confluency, the culture medium was replaced with that containing 5% EVs-depleted fetal bovine serum (VivaCell Biosciences. Certificate of analysis was shown in Supplementary Appendix I) and cultured for 48 h. The small EVs were extracted by standard differential centrifugation as follows: centrifugation at 3,000 g for 25 min at 4 °C and 10,000 g for 1 h at 4 °C to eliminate dead cells and cellular debris, followed by ultracentrifugation at 100,000 g for 3 h at 4 °C, and washes with phosphate-buffered saline (PBS). The small EVs identification was based on a general characterization standard of extracellular vesicles 19, 20. The morphology of EVs was observed with a transmission electron microscope (JEM-1011 Japan). The number and size were assessed using the NanoSight NS300 system (NanoSight, UK) following the manufacturer's protocol. The small EVs were identified by the EVs marker proteins CD9, CD63, ALIX, and cellular debris protein calnexin by Western blotting. The total protein concentration of EVs was measured by the BCA assay (ThermoScientific). The uptake of PKH67-labeled EVs was determined by confocal microscopy and flow cytometry.

### Animal experimental protocol

All animal experiments were approved by the Institutional Ethics Committee of Nanjing Drum Tower Hospital (Approval No. 20011141), and followed the guidelines outlined in the Guide for the Care and Use of Laboratory Animals published by the National Institutes of Health (Eighth Edition). C57BL/6 mice were purchased (8 weeks of age) from the Model Animal Research Center of Nanjing University. Animals were kept in temperature (22 ± 1 °C)- and humidity (65%-70%)-controlled room with a 12 h light-dark cycle, and fed a standard laboratory diet with free access to food and water. After the study, all animals were anesthetized using 1.5% isoflurane inhalation and then euthanized by cervical dislocation. Surgical induction of myocardial I/R was performed, as previously described 21. Mice underwent a period of ischemia for 45 min through ligating the left anterior descending (LAD) coronary artery and were followed by reperfusion. Immediately after reperfusion, the EV group mice were injected via caudal vein with a total of 100 μL PBS containing EVs at 3 μg/g of body weight. The dose of EVs was determined according to our preliminary dose-range experiment (Figure S1) and previously published studies 13, 16, 21. The artery was separated without deligation in the sham operation group. In macrophage depletion study, mice were intravenously injected with 150 μL (5 mg/ml) clodronate liposomes (Liposoma, The Netherlands) 1 day before and 1 day after myocardial I/R injury. In splenectomy study, the abdominal cavity was opened after isoflurane anesthesia, the spleen vessels were exposed, and cauterized with 4-0 silk suture, and the spleen was carefully removed 22. Seven days after splenectomy, myocardial I/R operation was performed.

### Statistical analysis

All values are presented as mean ± standard dEViation (SD) or median with interquartile ranges as appropriate. Comparisons between two groups were made using the Mann-Whitney U test, whereas the data obtained from multiple groups were compared using the Kruskal-Wallis test with Dunn's multiple comparison test or Bonferroni post hoc analysis. Two-way analysis of variance (ANOVA) followed by Bonferroni post hoc analysis was performed to analyze data with two factors. Differences with *p*-values < 0.05 were considered to be statistically significant. Statistical analyses were performed using GraphPad Prism 5.0 (Graph Pad Prism Software Inc., San Diego, CA, USA).

## Results

### Anti-inflammatory phenotype and KLF2 expression were maintained by laminar flow in cultured HUVECs

**Figure 1:**
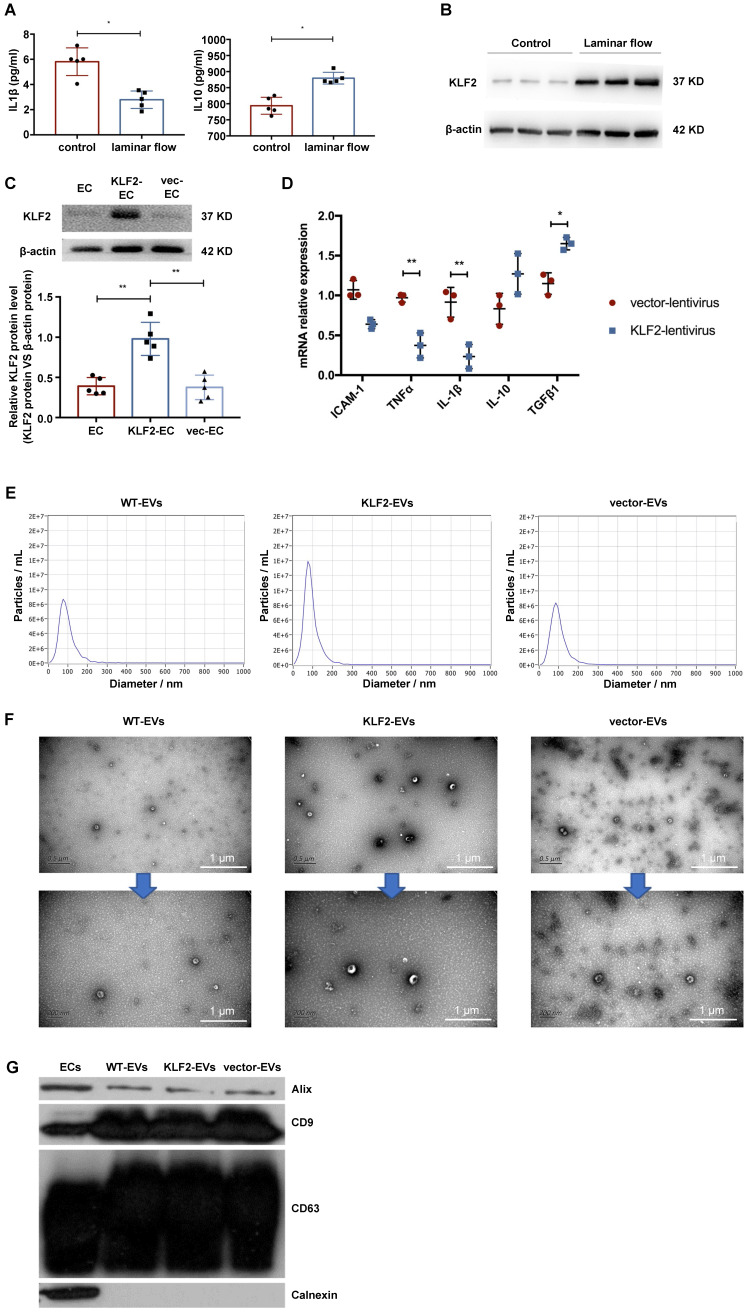
** HUVECs were activated into an anti-inflammatory phenotype by laminar shear stress with upregulation of KLF2, and characterization of extracellular vesicles (EVs) derived from KLF2-transduced HUVECs.** (**A**) Concentration of cytokines IL-1β and IL-10 detected using enzyme-linked immunosorbent assay (ELISA) in control and laminar shear stress group (n=5). (**B**) Representative images of Western blot (WB) to assess expression of KLF2 in control and laminar shear stress group (n=5). (**C**) Representative images of WB to assess expression of KLF2 in KLF2-transduced HUVECs among with empty vector-transduced HUVECs and untreated HUVECs (n=5). (**D**) Quantification of inflammatory cytokines with quantitative reverse transcriptase polymerase chain reaction (qRT-PCR), including IL-10, TGF-β1, ICAM-1, IL-1β and TNF-α in KLF2-transduced HUVECs and empty vector-transduced HUVECs (n=3). (**E**) Nanoparticle trafficking analyzed the diameters and concentration of WT-EVs, KLF2-EVs and vector-EVs. (**F**) Transmission electron micrograph of WT-EVs, KLF2-EVs and vector-EVs. Scale bar=0.5 µm/200 nm. (**G**) Representative images of WB to assess the presence of CD9, CD63, ALIX and Calnexin in EVs derived from wide type HUVECs (WT-EVs), EVs derived from KLF2-transduced HUVECs (KLF2-EVs), EVs derived from vector-transduced HUVECs (vector- EVs) and HUVECs. Graphs depict mean ± SD. Statistical significance was measured via Student's t-test for two groups' comparison, one-way ANOVA followed by Tukey's multiple comparisons test for multiple groups' comparison. *P < 0.05, **P < 0.01.

To confirm that KLF2 was activated by laminar shear stress and conferred its protective anti-inflammatory effects on the endothelium, we employed a parallel plate-type flow chamber and positive-displacement pump to drive the HUVECs culture medium. After 48 hours, we harvested cells and medium to assess inflammatory cytokines. As shown in Figure 1A and Figure S2A, laminar shear stress promoted anti-inflammatory cytokine interleukin (IL)-10 expression but decreased that of pro-inflammatory cytokines, including IL-1β. Furthermore, KLF2 was upregulated in HUVECs, both at mRNA and protein levels, under laminar shear stress (Figure 1B, Figure S2B-S2D). It has been reported that the laminar flow could maintain an anti-inflammatory and anti-thrombotic phenotype in endothelial cells by activating KLF2 in physiological vessels 23, 24. Hence, we explored whether overexpression of KLF2 could mimic the anti-inflammatory phenotype in HUVECs. We transduced HUVECs with a lentivirus vector encoding KLF2 (KLF2-HUVECs), resulting in a remarkable increase of KLF2 expression (Figure 1C, Figure S3). Next, we measured the expression of sEVeral cytokines with real-time PCR. Compared with empty vector-transduced HUVECs, KLF2 overexpression upregulated anti-inflammatory cytokines, including transforming growth factor (TGF)-β1, and downregulated pro-inflammatory cytokines, including IL-1β and tumor necrosis factor (TNF)-α (Figure 1D). These results indicated that HUVECs with upregulated KLF2 could mimic the anti-inflammatory phenotype of ECs under physiological laminar flow.

### EVs from KLF2-transduced HUVECs ameliorated myocardial I/R injury and alleviated inflammation

We used wide-type HUVECs (WT-HUVECs), KLF2-HUVECs, and vector-HUVECs, isolated extracellular vesicles (WT-EVs, KLF2-EVs, and vector-EVs) from the culture supernatants through ultracentrifugation, and characterized their phenotypes as previously described 19, 20. The nanoparticle tracking analysis (NTA) demonstrated that the diameters of the particles ranged from 50 to 150 nm, and the absolute numbers of particles were similar in three groups (Figure 1E). Besides, static and flow conditions did not influence the diameter and concentration of particles (Table S1 for detailed data, the analysis results are shown in Supplementary Appendix II). Transmission electron microscopy (TEM) showed that the particles had double-layer membrane structure and cup-shaped canonical EVs morphology, and were not affected by KLF2 overexpression (Figure 1F). Further, Western blot analysis confirmed that the particles expressed EVs makers such as CD63, CD9, and ALIX and were devoid of the cellular debris protein calnexin (Figure 1G). These properties validated the authenticity of isolated particles as EVs. Also, for the quantification of EVs, the protein concentration in particle fractions measured by BCA strongly correlated with particle number measured by NTA 21. Thus, we used BCA assay for the quantity of EVs in subsequent experiments.

**Figure 2:**
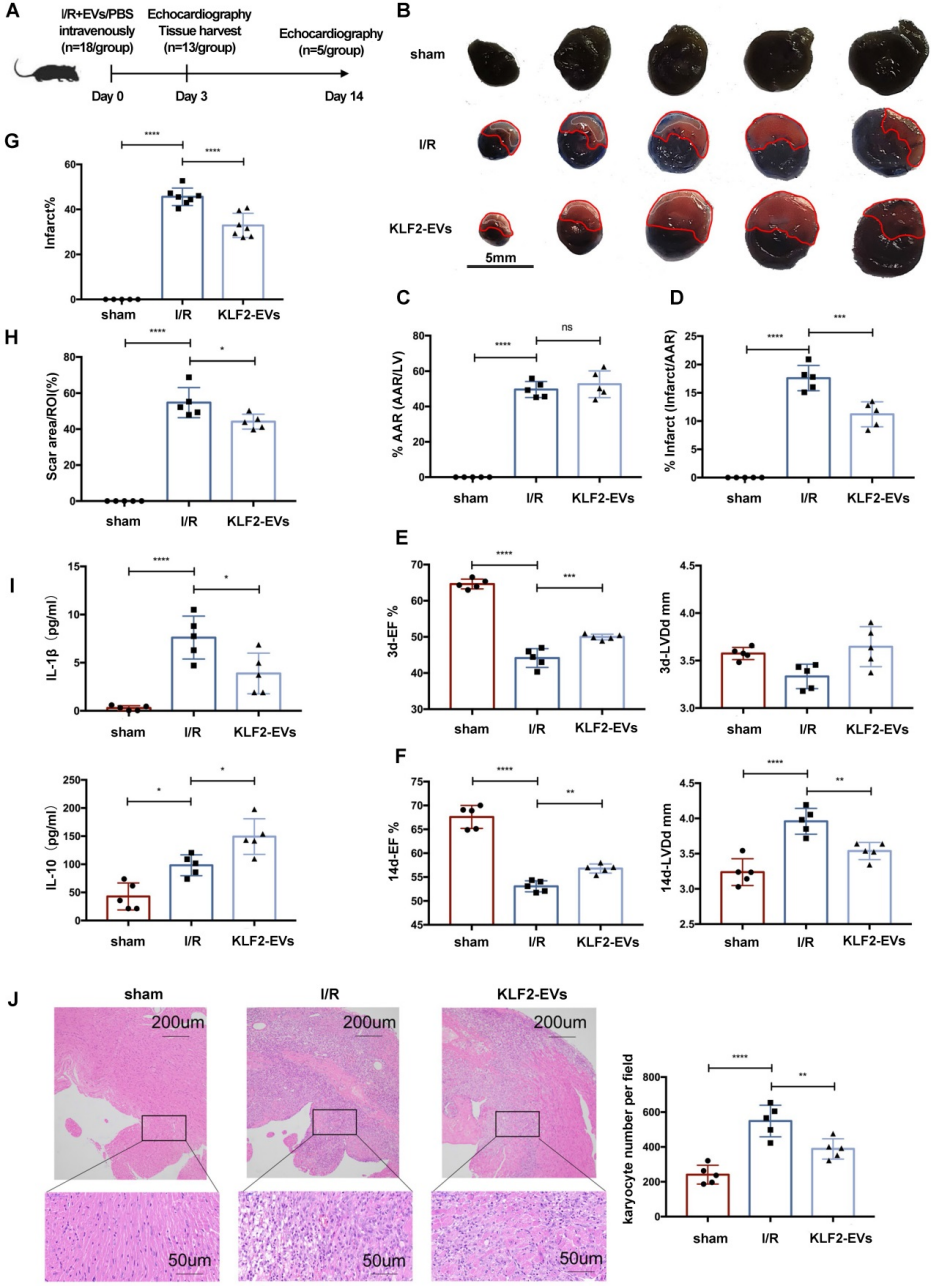
** Extracellular vesicles (EVs) from KLF2-transduced HUVECs ameliorated myocardial ischemia/reperfusion (I/R) injury and alleviated inflammation.** (**A**) Schematic of myocardial I/R injury model and intravenous infusion of EVs. (**B**) Representative images of hearts with Evans blue/TTC staining from mice 3 days following treatment with PBS or KLF2-EVs. Area-at-risk (AAR): red line; infarct size (IS): white dotted line. Scale bar=5 mm. Quantitative analysis of the percentage of AAR/left ventricle (**C**) and percentage of infarct/AAR (**D**) (n=5). Ejection fraction (EF) and left ventricular end-diastolic diameter (LVDd) of sham-operated, PBS treated or KLF2-EVs treated mice measured by echocardiography 3 days (**E**) and 2 weeks (**F**) following myocardial I/R injury (n=5). (**G**) Quantification of infarct area (%) in H.E. staining within the ischemic heart 3 days following operation (n=5 in sham group, n=7 in I/R or KLF2-EVs group). (**H**) Quantification of scar area (%) in Masson trichrome staining within the ischemic heart 2 weeks following operation (n=5). (**I**) Concentration of cytokines IL-1β and IL-10 detected by ELISA in serum of sham-operated, PBS or KLF2-EVs treated mice (n=5). (**J**) Representative images of H.E. staining and quantification of inflammatory cell infiltration (%) within the ischemic heart 3 days after operation (n=5). Scale bar=200 µm/50 µm. Graphs depict mean ± SD. Statistical significance was measured via one-way ANOVA followed by Tukey's multiple comparisons test for multiple groups' comparisons. *P < 0.05, **P < 0.01, ***P < 0.001, ****P < 0.0001, ns= not significant.

To investigate whether the KLF2-EVs ameliorated myocardial I/R injury, we surgically induced I/R injury in mice and injected KLF2-EVs into the caudal vein immediately after reperfusion; in the control I/R group, an equal volume of PBS was used (Figure 2A). First, we performed a preliminary dose-ranging experiment to evaluate different doses of KLF2-EVs contributing to ejection fraction (EF) and fractional shortening (FS), and selected 3μg/g of body weight as the best EVs dose (Figure S1). Next, the extent of cardiac I/R injury using Evans blue/TTC staining was determined on day 3 after reperfusion. With a similar size of areas at risk (AAR), the infarct area/AAR ratio was significantly lower in KLF2-EVs-treated mice than in control mice (Figure 2B-2D). Echocardiography (UCG) was used to assess cardiac function on day 3 and day 14 after myocardial I/R injury. Although cardiac function gradually improved over time in all I/R mice (Table S2 for detailed data), both EF and FS were increased on day 3 and 14 and left ventricular end-diastolic diameter (LVDd) was considerably decreased on day 14 in KLF2-EVs- treated mice compared with the I/R group (Figure 2E, 2F, Figure S4A-S4D), indicating improved cardiac function.

For histological assessment after UCG, we harvested the heart on day 3 and 14. Hematoxylin and Eosin staining on day 3 showed a significantly lower infarct area in KLF2-EVs-treated mice than in the I/R group (Figure 2G, Figure S4E). Masson trichrome staining on day 14 revealed that KLF2-EVs could also decrease scar area in the infarct zone (Figure 2H, Figure S4F). To elucidate the underlying mechanisms, we focused on inflammation-associated alterations. Reduced inflammatory cell infiltration was observed in KLF2-EVs-treated mice (Figure 2J), as well as decreased IL-1β and increased IL-10 levels in mice sera and heart tissues (Figure 2I, Figure S4G), indicating that KLF2-EVs could modulate inflammation following myocardial I/R injury. To confirm that KLF2 was crucial in mediating EVs' effect, we injected equivalent vector-EVs in mice after cardiac I/R injury and found that vector-EVs could not reduce infarct size (Figure S5A-S5C), improve cardiac function in UCG (Figure S5D-S5F, Table S3 for detailed data), or suppress inflammation (Figure S5G, S5H). Therefore, we further explored the underlying mechanism of KLF2-EVs' effect on myocardial I/R injury.

### KLF2-EVs reduced inflammation level after I/R injury by decreasing Ly6C^high^ monocytes and systemic depletion of monocytes/macrophages obliterated the efficacy of KLF2-EVs therapy

**Figure 3:**
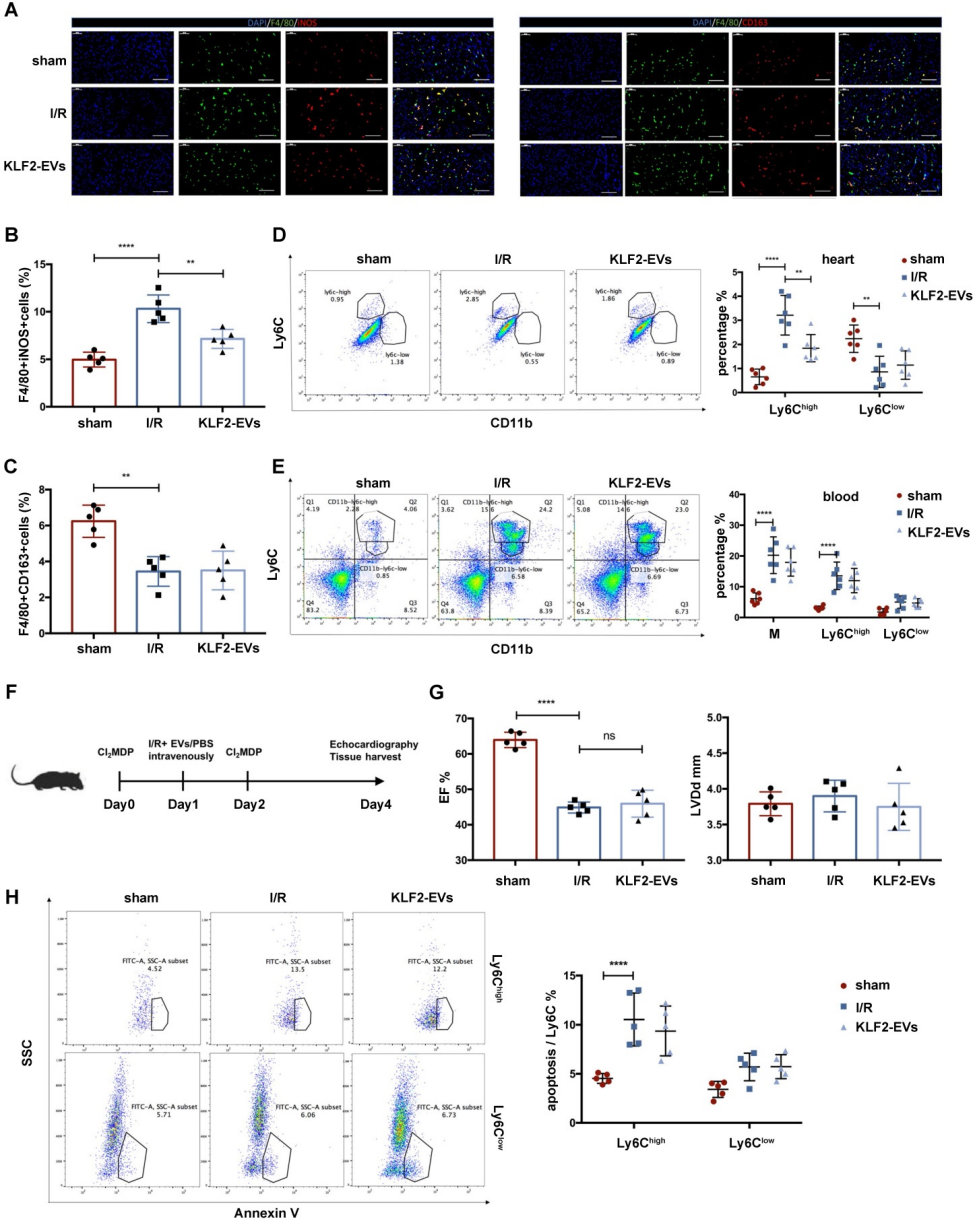
** KLF2-EVs reduced inflammation level after I/R injury by decreasing Ly6C^high^ monocytes.** (**A**) Representative immunofluorescence staining of Ly6C^high^ phenotype (F4/80+iNOS+) and Ly6C^low^ phenotype (F4/80+CD163+) in hearts of sham-operated, PBS or KLF2-EVs treated mice and quantification of double positive cells proportion in F4/80+ cells (**B, C**) 3 days following I/R injury (n=5). Scale bar= 50 µm. (**D**) Representative flow cytometry plots showing Ly6C^high^ monocyte/macrophage (Mo/Mø) (CD11b+Ly6C^high^) and Ly6C^low^ Mo/Mø (CD11b+Ly6C^low^), and quantification of cells within heart tissue 3 days following treatment (n=6). (**E**) Representative flow cytometry plots showing total monocytes, Ly6C^high^ monocytes (CD11b+Ly6C^high^) and Ly6C^low^ monocytes (CD11b+Ly6C^low^), and quantification of cells in peripheral blood 3 days following treatment (n=6). (**F**) Schematic of Mo/Mø depletion protocol with Cl_2_MDP liposomes. (**G**) Ejection fraction (EF) and left ventricular end-diastolic diameter (LVDd) of sham-operated, PBS or KLF2-EVs treated mice measured by echocardiography 3 days after myocardial I/R injury combined with systemic depletion of endogenous Mo/Mø (n=5). (**H**) Representative flow cytometry plots showing apoptosis of monocytes, primary gate is Ly6C^high^ Mo/Mø (CD11b+Ly6C^high^) or Ly6C^low^ Mo/Mø (CD11b+Ly6C^low^) in heart tissue 3 days following treatment, and quantification of Annexin V+ cells proportion in primary gate (n=5). Graphs depict mean ± SD. Statistical significance was measured via one-way ANOVA followed by Tukey's multiple comparisons test for multiple groups' comparison and two-way ANOVA followed by Bonferroni's multiple comparisons test for comparison between different groups in different cell subtypes. **P < 0.01, ****P < 0.0001, ns= not significant.

Because monocytic cells are a key mediator in post-I/R injury wound healing, we hypothesized that KLF2-EVs might modulate monocyte and macrophage responses. Since two Mo/Mø subsets (Ly6C^high^ and Ly6C^low^) are implicated in recovery after I/R injury, we examined the alteration of these two types of Mo/Mø. Immunofluorescence staining revealed that F4/80^+^ iNOS^+^ cells (Ly6C^high^ Mø) were significantly decreased, but F4/80^+^ CD163^+^ cells (Ly6C^low^ Mø) had no difference in the border zone surrounding the infarct area (Figure 3A-C). Besides, flow cytometry analysis of heart tissues harvested on day 3 after I/R injury showed significantly reduced Ly6C^high^ Mo/Mø in KLF2-EVs-treated mice but there was no change in Ly6C^low^ Mo/Mø (Figure 3D). We analyzed two types of monocytes in peripheral blood 3 days after the operation and found that KLF2-EVs did not alter the total Ly6C^high^ and Ly6C^low^ monocytes in the blood (Figure 3E).

Further, we investigated whether Mo/Mø played an exclusive role in the KLF2-EVs-mediated effect. We administered clodronate (dichloromethylene diphosphonate, Cl_2_MDP) liposomes 24 hours before and after myocardial I/R to systemically deplete Mo/Mø, and PBS or KLF2-EVs were injected through tail vein immediately after reperfusion (Figure 3F). As expected, Cl_2_MDP significantly reduced blood and spleen monocyte populations, as determined by flow cytometry analysis, and Mo/Mø population showed a descending tendency but with no statistical significance (*P*>0.05) (Figure S6A). However, with systemic depletion of Mo/Mø, KLF2-EVs lost the therapeutic effect of improving cardiac function (Figure 3G, Figure S6B, S6C & Table S4 for detailed data). These results indicated that the potential effects of KLF2-EVs were ascribed to Mo/Mø quantity and subpopulation.

### KLF2-EVs inhibited Ly6C^high^ monocyte recruitment from bone marrow (BM)

Since Ly6C^high^ Mo/Mø were decreased in the heart of KLF2-EVs-treated mice, we determined KLF2-EVs' effect on apoptosis and polarization of Ly6C^high^ Mo/Mø. KLF2-EVs did not change the proportion of Annexin V+ cells for early-stage apoptosis in two Mo/Mø subgroups *in vivo* after myocardial I/R (Figure 3H). Similar results were obtained *in vitro* when RAW264.7 cells were subjected to hypoxia/reoxygenation treatment (Figure S7A). Besides, we found that KLF2-EVs could not polarize macrophages from M1 to M2 phenotype under lipopolysaccharide (LPS) stimulation (Figure S7B). These results indicated that KLF2-EVs did not affect the apoptosis and polarization of Ly6C^high^ Mo/Mø. Thus, we speculated that impaired Ly6C^high^ monocyte recruitment might contribute to decreased Ly6C^high^ Mo/Mø in heart tissue after KLF2-EVs administration.

**Figure 4:**
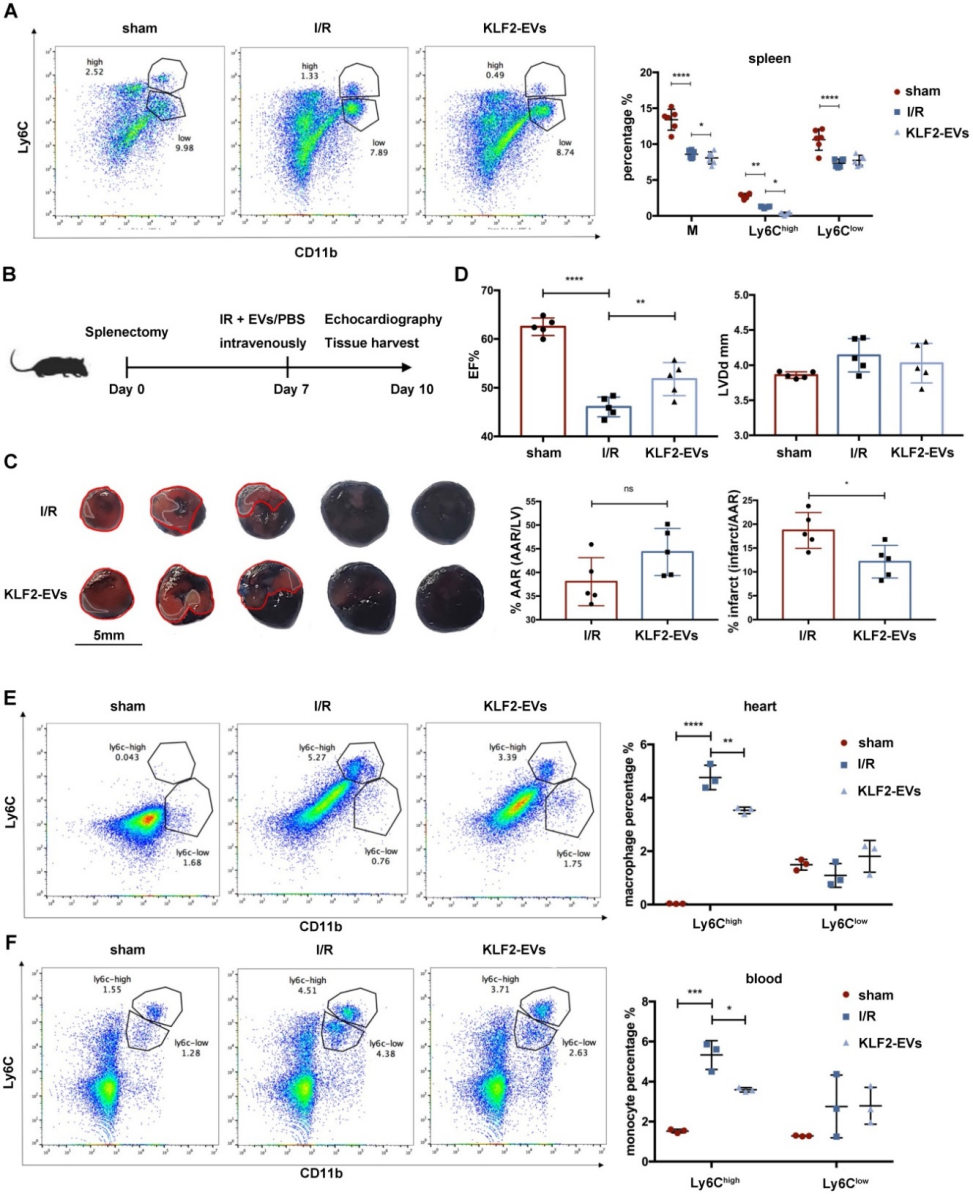
** KLF2-EVs did not inhibited Ly6C^high^ monocytes recruitment from spleen.** (**A**) Representative flow cytometry plots showing total monocytes, Ly6C^high^ monocytes (CD11b+Ly6C^high^) and Ly6C^low^ monocytes (CD11b+Ly6C^low^), and quantification of cells in spleen 3 days following treatment (n=6). (**B**) Schematic of splenectomy protocol followed by myocardial ischemia/reperfusion (I/R) injury. (**C**) Representative images of hearts with Evans blue/TTC staining from mice 3 days following treatment with PBS or KLF2-EVs. Area-at-risk (AAR): red line; infarct size (IS): white dotted line. Scale bar=5 mm. Quantitative analysis of the percentage AAR and percentage infarct of hearts (n=5). (**D**) Ejection fraction (EF) and left ventricular end-diastolic diameter (LVDd) of sham-operated, PBS or KLF2-EVs treated mice measured by echocardiography 3 days following myocardial I/R injury combined with splenectomy (n=5). Representative flow cytometry plots showing Ly6C^high^ Mo/Mø (CD11b+Ly6C^high^) and Ly6C^low^ Mo/Mø (CD11b+Ly6C^low^) and quantification of cells within heart tissues (**E**) or peripheral blood (**F**) 3 days following treatment (n=3). Graphs depict mean ± SD. Statistical significance was measured via Student's *t*-test for two groups' comparison, one-way ANOVA followed by Tukey's multiple comparisons test for multiple groups' comparison and two-way ANOVA followed by Bonferroni's multiple comparisons test for comparison between different groups in different cell subtypes. *P < 0.05, **P < 0.01, ***P < 0.001, ****P < 0.0001, ns = not significant.

It is widely believed that in response to STEMI, monocytes originate from the spleen and BM, and the spleen is a major site of monocyte production after MI during the first two days 25. We hypothesized that KLF2-EVs might interdict Ly6C^high^ monocyte recruitment from the spleen, and, therefore, splenectomy might block the therapeutic efficacy. We first examined Ly6C^high^ and Ly6C^low^ monocytes in the spleen after myocardial I/R injury and found the Ly6C^high^ monocyte reservoir in the spleen to be lower in KLF2-EVs group than in the I/R group (Figure 4A). Then, the spleen was removed 7 days before I/R, and cardiac function and the infarct area was evaluated 3 days after I/R (Figure 4B). Evans blue/TTC staining results illustrated that the infarct area was smaller and, as per echocardiography, cardiac function was better in the KLF2-EVs group than in the I/R group (Figure 4C, 4D, Figure S8A, S8B & Table S5 for detailed data). Besides, when we compared sham group and splenectomy group, I/R group and I/R+splenectomy group or I/R+KLF2-EVs group and I/R+splenectomy+KLF2-EVs group, we found cardiac function was not altered after splenectomy (Figure S8C & S8D). Meanwhile, Ly6C^high^ Mo/Mø in the heart (Figure 4E) and Ly6C^high^ monocytes in the blood were decreased (Figure 4F). A previous study reported that the spleen contributes 40-75% monocytes to the ischemic myocardium by Atgr1 interacted with Ang II 25. Therefore, we analyzed Atgr1 in the spleen and found no alteration in Atgr1 mRNA and protein lEVels among different treatment groups (Figure S9A, S9B). These data suggested that the protective effect of KLF2-EVs was not altered after splenectomy.

**Figure 5:**
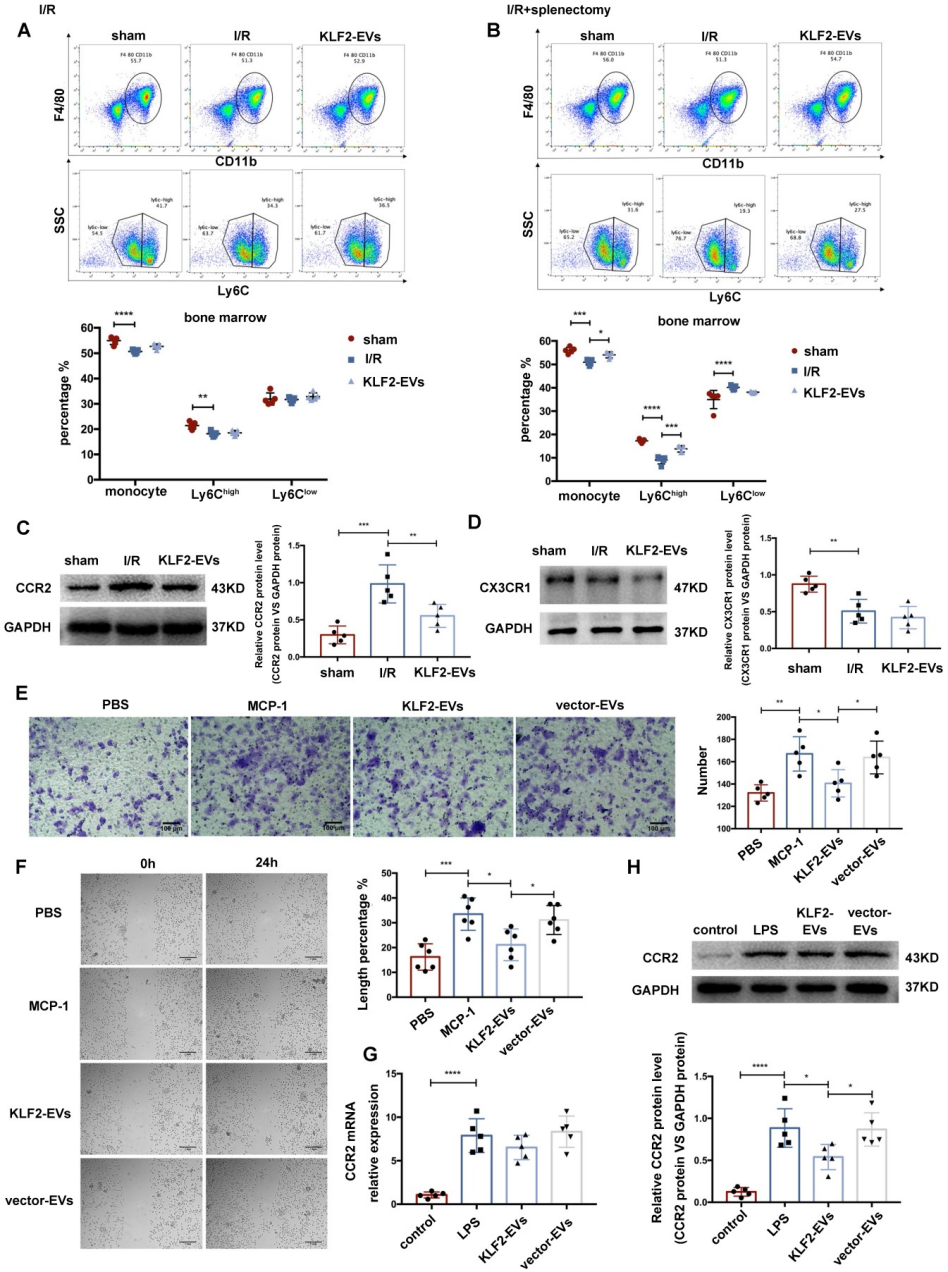
** KLF2-EVs inhibited Ly6C^high^ monocytes recruitment from bone marrow *in vivo* and Mo/Mø migration *in vitro*.** (**A**) Representative flow cytometry plots showing total monocyte, Ly6C^high^ monocyte (CD11b+Ly6C^high^) and Ly6C^low^ monocyte (CD11b+Ly6C^low^), and quantification of cells in bone marrow (BM) 3 days following PBS or KLF2-EVs treatment (n=5). (**B**) Representative flow cytometry plots showing total monocyte, Ly6C^high^ monocyte and Ly6C^low^ monocyte, and quantification of cells in BM 3 days following PBS or KLF2-EVs treatment with splenectomy (n=5). Representative images of WB and quantification to assess expression of CCR2 (**C**) and CX3CR1 (**D**) within BM in sham-operated, PBS and KLF2-EVs groups (n=5). (**E**) Representative images of BMDMs in Transwell experiment with treatment of EVs and MCP-1, and quantification of number of migrated cells (n=5). Scale bar=100 µm. (**F**) Representative images of BMDMs in scratch wound healing assay with treatment of EVs and MCP-1, and quantification of length percentage of cells migration (n=5). Scale bar=1 mm. (**G**) Quantification of mRNA of CCR2 of BMDMs using qRT-PCR in control, LPS, KLF2-EVs and vector-EVs groups (n=5). (**H**) Representative images of WB and quantification to assess expression of CCR2 of BMDMs in control, LPS, KLF2-EVs and vector-EVs groups (n=5). Graphs depict mean ± SD. Statistical significance was measured via one-way ANOVA followed by Tukey's multiple comparisons test for multiple groups' comparison and two-way ANOVA followed by Bonferroni's multiple comparisons test for comparison between different groups in different cell subtypes. *P < 0.05, **P < 0.01, ***P < 0.001, ****P < 0.0001.

The post-splenectomy results prompted us to hypothesize that KLF2-EVs were likely to regulate BM monocyte kinetics. We, therefore, investigated BM monocytes after administering KLF2-EVs without splenectomy and found no significant difference in total monocytes, Ly6C^high^ or Ly6C^low^ monocytes between the KLF2-EVs and I/R groups (Figure 5A). However, upon splenectomy, monocytes and the Ly6C^high^ monocyte population in the BM were higher in the KLF2-EVs group than in the I/R group (Figure 5B). These results indicated that KLF2-EVs might inhibit the Ly6C^high^ monocytes recruited from BM. This effect became obvious upon splenectomy.

Next, we examined the expression of CCR2 and CX3CR1 associated with monocyte activation in I/R mice without splenectomy. qRT-PCR and Western blotting were performed to measure CCR2 and CX3CR1 expression in the BM. CCR2 and CX3CR1 were reported to participate in Ly6C^high^ and Ly6C^low^ monocyte activation, respectively. The mRNA levels of CCR2 and CX3CR1 were not altered between the KLF2-EVs and I/R groups (Figure S9C), but the expression of CCR2 protein was reduced in KLF2-EVs-treated mice (Figure 5C) while the expression of CX3CR1 protein remained unchanged (Figure 5D). A similar phenomenon was detected by immunohistochemical staining of the heart tissue (Figure S9D). We performed ELISA to investigate whether MCP-1 (also known as CCL2, the ligand of CCR2) and fractalkine (also known as CX3CL1, the ligand of CX3CR1) were altered in mice sera, and found no significant difference (Figure S9E). These results illustrated that Ly6C^high^ monocyte recruitment from BM was inhibited by KLF2-EVs treatment.

### KLF2-EVs restrained Mo/Mø migration *in vitro*

We explored the effect of KLF2-EVs on Mo/Mø *in vitro*. The Transwell and scratch wound healing assays were performed to detect migration of primary BM-derived macrophages (BMDMs) and RAW264.7 cells under MCP-1 induction. We extracted primary BMDMs from mice BM and cultured them for seven days with M-CSF as previously described 26. KLF2-EVs inhibited the migration of BMDMs, resulting in reduced cell numbers (Figure 5E) and larger scratch size (Figure 5F) compared with the MCP-1 and vector-EVs groups. Furthermore, as in the animal experiments, KLF2-EVs decreased CCR2 protein expression (Figure 5H) but did not affect the mRNA level (Figure 5G) compared with the LPS and vector-EVs groups. Similar results were obtained for RAW264.7 cells in which KLF2-EVs inhibited their migration compared with the MCP-1 and vector-EVs groups (Figure S10A, S10B), and reduced CCR2 protein expression compared with the LPS group (Figure S10C, S10D). In contrast, CX3CR1 expression was not affected (Figure S10E, S10F). These results indicated that KLF2-EVs might be involved in the post-transcriptional regulation of the CCR2-mediated Ly6C^high^ Mo/Mø migration.

**Figure 6:**
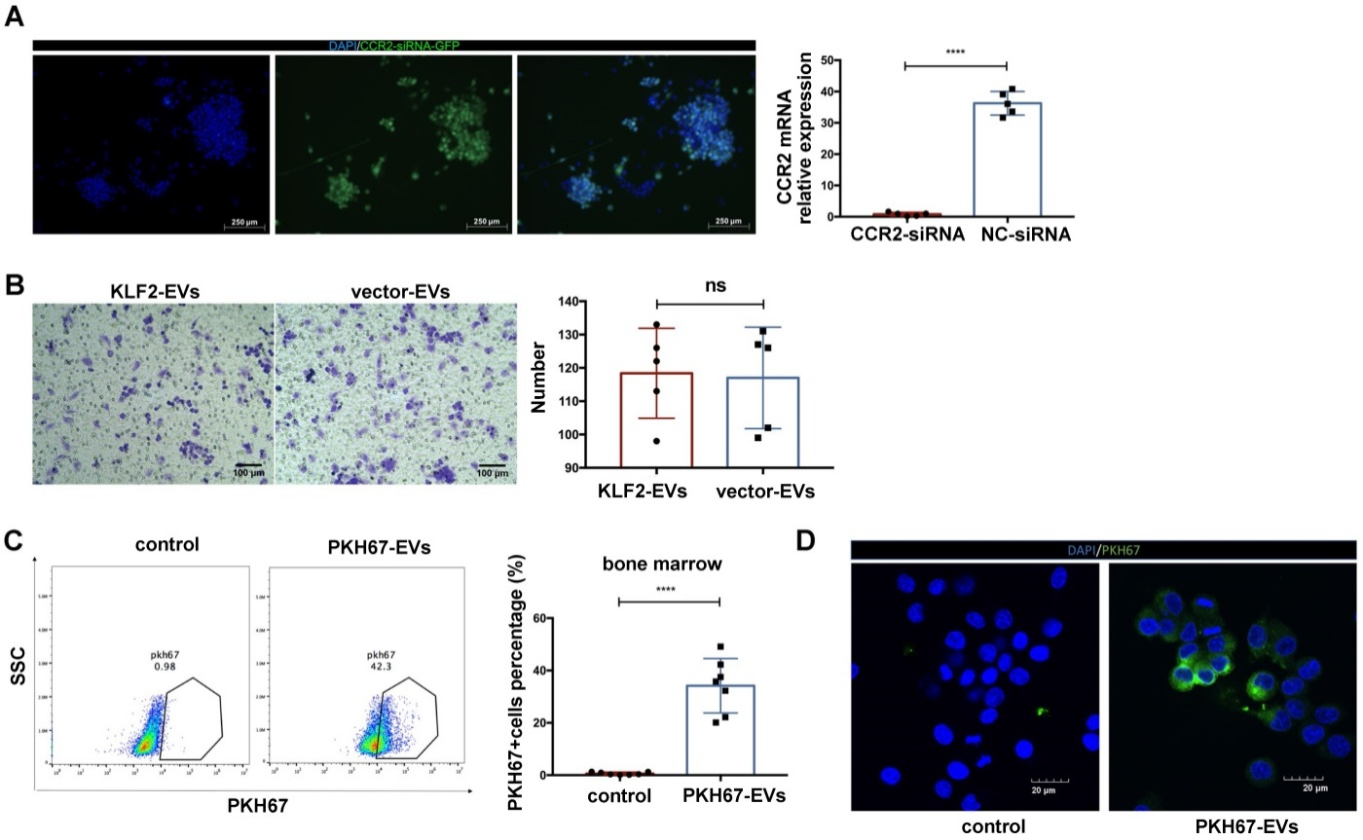
** The deletion CCR2 in BMDMs abrogated the effect of KLF2-EVs on inhibiting BMDMs migration, and KLF2-EVs were localized in BM and internalized by macrophages.** (**A**) Representative fluorescence staining of CCR2-siRNA (GFP green) in BMDMs. Scale bar=250 µm. Quantification of mRNA of CCR2 using qRT-PCR in CCR2-siRNA transduced BMDMs and NC-siRNA transduced BMDMs (n=5). (**B**) Representative images of BMDMs in Transwell experiment with treatment of KLF2-EVs or vector-EVs derived from BMDMs, and quantification of number of migrated cells (n=5). Scale bar=100 µm. (**C**) Representative flow cytometry plots and quantification showing percentage of cells in BM that absorbed PKH67-labeled EVs (n=7). (**D**) Representative images of the uptake of PKH67-labeled exosomes (green) by RAW264.7 cells (DAPI blue) and fluorescence uptake with PKH67 dye sample. Graphs depict mean ± SD. Statistical significance was measured via Student's *t*-test for two groups' comparison. ****P < 0.0001, ns = not significant.

To confirm that the CCR2 protein was the target of KLF2-EVs, we deleted CCR2 in BMDMs through small interfering RNA (siRNA) (Figure 6A), which blocked KLF2-EVs' effect on inhibiting BMDMs migration (Figure 6B).

### KLF2-EVs were localized in BM and internalized by macrophages

We explored whether small EVs localized to BM and disrupted monocyte activation. EVs were labeled with a fluorescent membrane marker PKH67 (green), and injected intravenously into I/R injury mice. After 24 h, 34.19% cells PKH67‐labeled EVs were detected in BM compared with the PKH67 dye immediately after injecting into mice serving as the control (Figure 6C).

Further, to confirm the small EVs could be internalized by the macrophages *in vitro*, the PKH67‐labeled EVs were added to the culture medium of RAW264.7 cells. After 12 h of incubation, PKH67‐labeled EVs were taken up by macrophages and localized in the cytoplasm compared with cells immediately after adding PKH67 (Figure 6D).

### miR-24-3p regulated KLF2-EVs-mediated Ly6C^high^ monocyte recruitment

**Figure 7:**
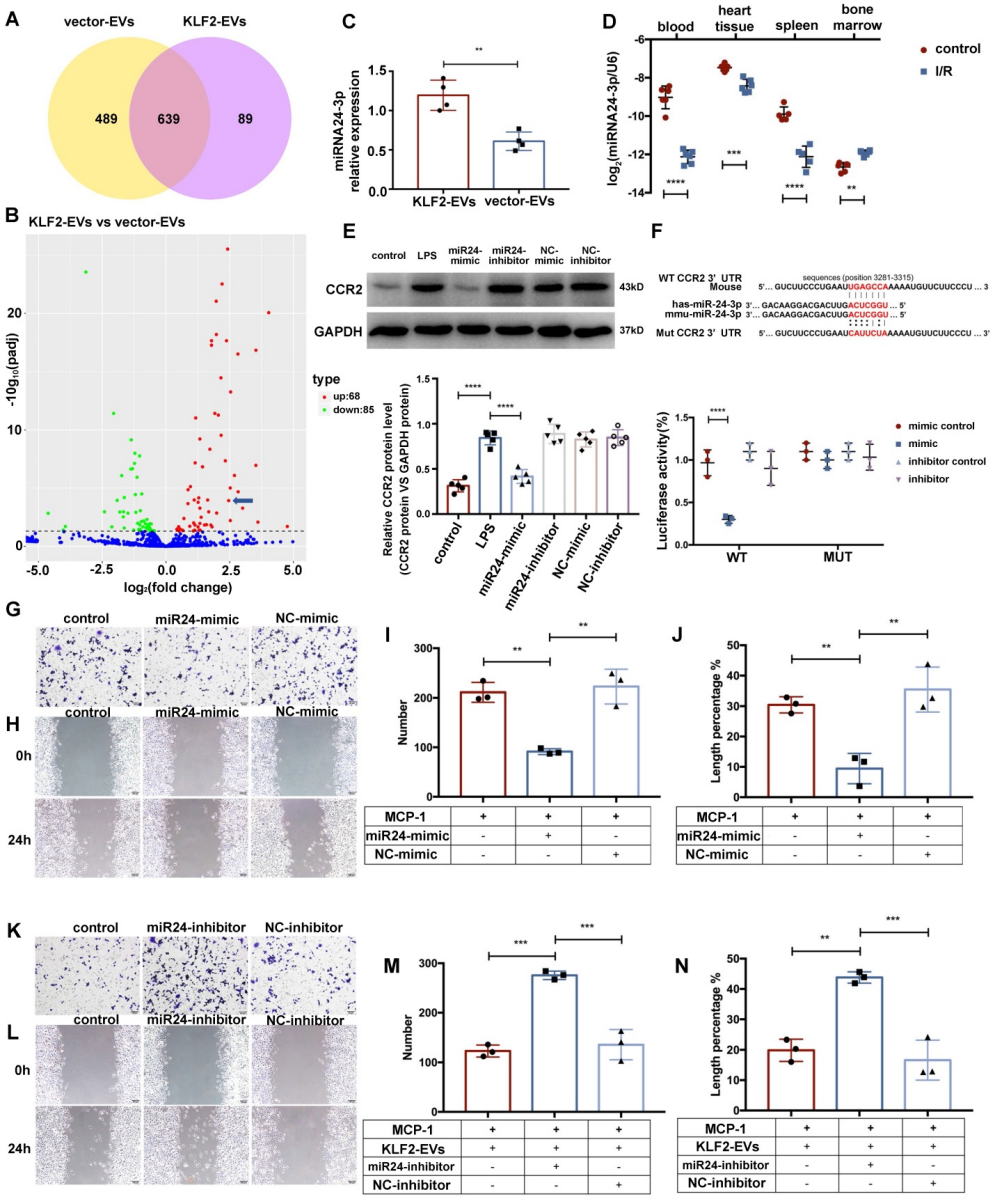
** miR-24-3p regulated KLF2-EV-mediated Ly6C^high^ monocyte recruitment.** (**A**) Venn plot achieved from miRNA microarray. 489 miRNAs were detected only in KLF2-EVs (yellow cycle), and 89 miRNAs were detected only in vector-EVs (purple cycle) while 639 miRNAs in common. (**B**) Volcano plot showing the level of expression of the differentially expressed miRNAs from KLF2-EVs compared to vector-EVs. The blue arrow pointed miR-24-3p [log2(fold change) = 2.4611, -log10 (*p*adj) = 3.93]. (**C**) Quantification of miR-24-3p in KLF2-EVs compared to vector-EVs (n=4). (**D**) Quantification of miR-24-3p in different tissues before and after myocardial I/R injury (n=6). (**E**) Representative images of WB and quantification to assess expression of CCR2 in treatment of control, LPS, miR-24-3p or NC mimic/inhibitor (n=5). (**F**) Luciferase reporter containing the wild-type or mutated 3′-UTR segment of mouse CCR2 mRNA. The sequence in red indicates the predicted binding site for human or mouse miR-24-3p and CCR2 mRNA. Quantification of relative luciferase activity normalized to control (n=3). Representative images of RAW264.7 cells in Transwell experiment (**G**, scale bar=50 µm) and scratch wound healing assay (**H**, scale bar=100 µm) with treatment of miR-24-3p mimic and MCP-1. Quantification of number of migrated cells (**I**) and length percentage of cells migration (**J**) (n=3). Representative images of RAW264.7 cells in Transwell experiment (**K**, scale bar=50 µm) and scratch wound healing assay (**L**, scale bar=100 µm) with treatment of miR-24-3p inhibitor, KLF2-EVs and MCP-1. Quantification of number of migrated cells (**M**) and length percentage of cells migration (**N**) (n=3). Graphs depict mean ± SD. Statistical significance was measured via Student's *t*-test for two groups' comparison, one-way ANOVA followed by Tukey's multiple comparisons test for multiple groups' comparison. **P < 0.01, ***P < 0.001, ****P < 0.0001.

Accumulating evidence suggests that EVs transfer signals between cells via bioactive molecules, including miRNAs 15. We explored whether KLF2-EVs contained any specific miRNAs accounting for their immunomodulatory effect. Following miRNA array analysis and normalization of raw sequencing data, 728 and 1128 miRNAs were detected in KLF2-EVs and vector-EVs. Among these, 639 miRNAs were common (Figure 7A), and 153 miRNAs were differentially expressed with 68 miRNAs showing upregulated (Table S6) and 85 downregulated expression (Figure S11A). We selected 23 miRNAs that had significantly higher expression (log2FC > 2) in KLF2-EVs than vector-EVs. According to absolute enrichment (KLF2_EVs_readcount > 10000), 4 miRNAs (hsa-miR-24-3p, hsa-let-7a-5p, hsa-let-7f-5p and hsa-miR-125b-5p) were abundant in KLF2-EVs. Next, we analyzed the potential target genes of 4 miRNAs in GO pathway database (Figure S11B) and found only miR-24-3p was related to negative regulation of cells migration. Based on comprehensive functional enrichment analysis of target genes (data not shown) and previously published studies 27-29, miR-24-3p, a highly conserved miRNA with homologous sequences in human and mouse, was selected for further exploration (Figure 7B). qRT-PCR confirmed that miR-24-3p was more abundant in KLF2-EVs than vector-EVs (Figure 7C). We further quantified the constitutive expression of miR-24-3p in myocardial I/R mice. Interestingly, compared to mice without I/R, miR-24-3p was decreased in peripheral blood, heart tissue, and spleen in mice post-I/R but dramatically increased in BM (Figure 7D).

Using TargetScan7.1 and mirdbv5, the potential target genes of the miR-24-3p were predicted. Results indicated the CCR2 gene, essential for cell migration, as a potential target for miR-24-3p. We performed a dual-luciferase assay to assess the interaction between miR-24-3p and the CCR2 gene and found the relative activity of luciferase was lowest in cells co-transfected with the plasmid carrying CCR2 3'UTR sequences and miR-24-3p mimics while the luciferase activity in cells transfected with the mutant CCR2 3'UTR sequence and miR-24-3p mimics remained unchanged (Figure 7F). These results suggested specific binding of miR-24-3p to the CCR2 mRNA 3'UTR sequence.

The regulatory effect of miR-24-3p on CCR2 expression was verified in RAW264.7 cells incubated with KLF2-EVs carrying miR-24-3p mimic or miR-24-3p inhibitor (Figure S11C). The results showed that miR-24-3p mimic attenuated the migration of monocytes both in the Transwell experiment and scratch wound healing assay under the inflammatory environment compared with the NC mimic (Figure 7G-7J), whereas miR-24-3p inhibitor abrogated the effect of KLF2-EVs (Figure 7K-7N). Also, miR-24-3p mimic decreased the expression of CCR2 protein (Figure 7E) but did not influence its mRNA level (Figure S11D). These results demonstrated that miR-24-3p was the key regulatory component in KLF2-EVs that contributed to monocyte recruitment regulation.

### miR-24-3p antagomir abrogated the effect of KLF2-EVs on ameliorating myocardial I/R injury

**Figure 8:**
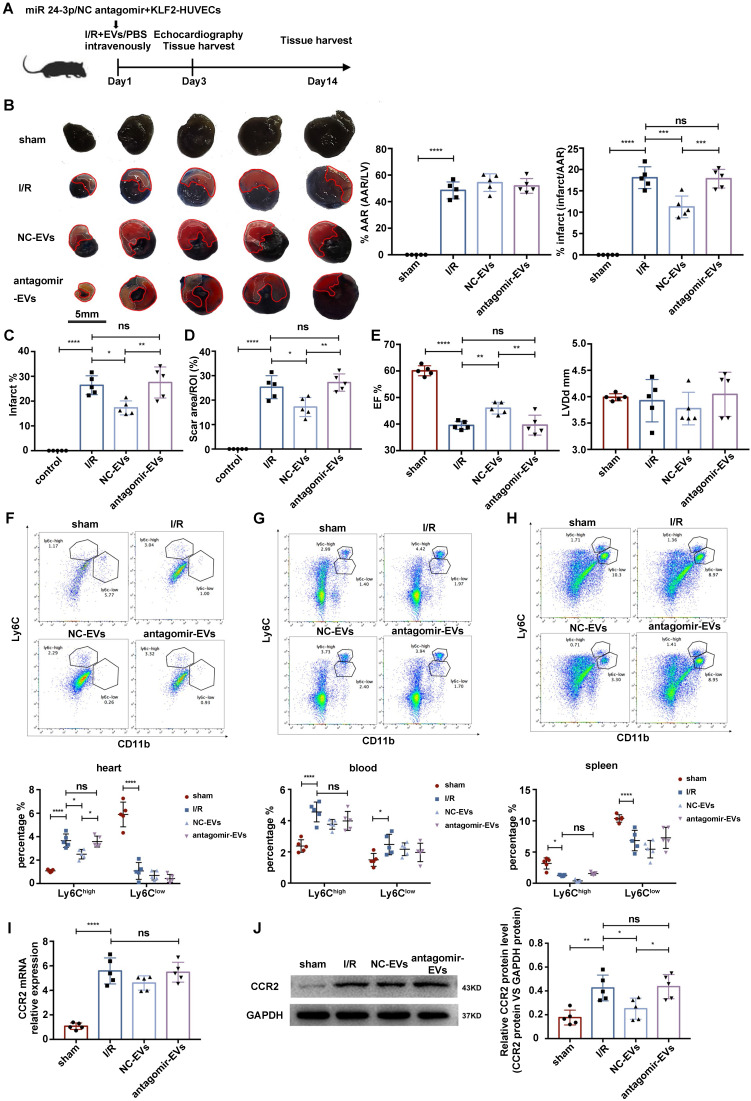
** miR-24-3p antagomir abrogated the effect of KLF2-EVs on ameliorating myocardial I/R injury.** (**A**) Schematic of myocardial I/R injury model and intravenous infusion of EVs derived from miR-24-3p antagomir or NC antagomir treated HUVECs. (**B**) Representative images of hearts in Evans blue/TTC staining from mice 3 days following treatment with PBS, NC-EVs or antagomir-EVs. Area-at-risk (AAR): red line; infarct size (IS): white dotted line. Scale bar=5 mm. Quantitative analysis of the percentage AAR and percentage infarct of hearts (n=5). (**C**) Quantification of infarct area (%) in H.E. staining within the ischemic heart 3 days following operation (n=5). (**D**) Quantification of scar area in I/R hearts with Masson trichrome staining (n=5). (**E**) Ejection fraction (EF) and left ventricular end-diastolic diameter (LVDd) of differently treated mice measured by echocardiography 3 days following myocardial I/R injury (n=5). Representative flow cytometry plots showing Ly6C^high^ Mo/Mø (CD11b+Ly6C^high^) and Ly6C^low^ Mo/Mø (CD11b+Ly6C^low^), and quantification of cells within heart tissues (**F**), in peripheral blood (**G**) and in spleen (**H**) 3 days following treatment (n=5). (**I**) Quantification of CCR2 mRNA of BM with qRT-PCR in sham-operated, I/R, NC-EVs and antagomir-EVs groups (n=5). (**J**) Representative images of WB and quantification to assess expression of CCR2 within BM in sham-operated, I/R, NC-EVs and antagomir-EVs groups (n=5). Graphs depict mean ± SD. Statistical significance was measured via one-way ANOVA followed by Tukey's multiple comparisons test for multiple groups' comparison and two-way ANOVA followed by Bonferroni's multiple comparisons test for comparison between different groups in different cell subtypes. *P < 0.05, **P < 0.01, ***P < 0.001, ****P < 0.0001, ns= not significant.

To assess whether the cardioprotective effects of KLF2-EVs depended on miR-24-3p, we applied miR-24-3p antagomir or negative control (NC) antagomir to KLF2-HUVECs and purified the EVs from the supernatants of cultured cells (antagomir-EVs and NC-EVs). The results showed decreased miR-24-3p concentration by the miR-24-3p antagomir (Figure S12A). Next, we injected the EVs intravenously into mice immediately after myocardial I/R injury and compared the extent of cardiac I/R injury and cardiac function between antagomir-EVs- and NC-EVs-treated mice on day 3 and day 14 after reperfusion (Figure 8A). We found a reduction in the infarct area/AAR ratio by Evans blue/TTC staining (Figure 8B), alteration of infarct area by H.E. staining (Figure 8C & Figure S12B), and the scar area (Figure 8D & Figure S12C) in Masson trichrome staining was recovered by miR-24-3p antagomir administration. Besides, UCG data illustrated improvement of cardiac function, such as EF, FS, and LVDd (Figure 8E, Figure S12D, S12E & Table S7 for detailed data), on day 3 after myocardial I/R injury caused by KLF2-EVs was reversed by miR-24-3p antagomir but not NC antagomir application. In addition, the decrease of Ly6C^high^Mo/Mø in heart tissue was abated by miR-24-3p antagomir pre-treatment (Figure 8F-H). The expression of CCR2 mRNA and protein in BM was unchanged in antagomir-EVs-treated mice compared to I/R mice but the CCR2 protein expression decreased in NC-EVs-treated mice (Figure 8I, J). These data indicated that miR-24-3p recapitulated the benefits of KLF2-EVs in myocardial I/R injury.

### KLF2-MCAECs-derived EVs ameliorated myocardial I/R injury and were rich in miR-24-3p

The EVs containing functional biomolecules are considered advantageous for clinical use because of their low immunogenicity; hence HUVECs EVs were used in this study to assess their protective effect in cardiac I/R injury. To avoid species differences, we obtained MCAECs and observed the therapeutic role of KLF2-MCAECs-derived EVs. First, we characterized Dil-AcLDL uptake by MCAECs and staining with endothelial cells surface marker CD31 to test the purity of the population. Almost all cells expressed CD31 and took in the Dil-AcLDL (Figure S13A & S13B), indicating the successful isolation of MCAECs. Next, we transduced KLF2-lentivirus into MCAECs, resulting in a remarkable increase of KLF2 expression (Figure S13C-S13E). The EVs were purified from the culture supernatants and intravenously injected into mice immediately after reperfusion. Evans blue/TTC staining indicated that the infarct area was smaller (Figure S13F) and echocardiography results showed better cardiac function after KLF2-overexpressing MCAECs-derived EVs treatment (Figure S13G & Table S8 for detailed data). Thus, KLF2-EVs originating from mouse endothelial cells ameliorated myocardial I/R injury and allEViated inflammation level, indicating that effectors in EVs were highly conserved. Finally, we detected miR-24-3p in MCAECs-derived EVs and found it to be more abundant in KLF2-EVs than vector-EVs (Figure S13H) that likely served as the KLF2-EVs effector in MCAECs.

## Discussion

I/R injury following STEMI is still a critical issue without effective therapy in clinical practice. An optimal inflammatory cascade after I/R injury is thought to be beneficial for ischemic myocardium repair and better prognosis. In this study, we first highlighted the potential therapeutic effects of KLF2-EVs in immunomodulation following myocardial I/R injury. Subsequently, we established the Mo/Mø depletion model and performed splenectomy to show that Ly6C^high^ monocyte recruitment from BM was the main target of KLF2-EVs. Furthermore, we demonstrated that miR-24-3p was abundant in KLF2-EVs, and inhibited Ly6C^high^ Mo/Mø migration by post-transcriptionally decreasing CCR2 expression. We found miR-24-3p antagomir could abrogate the effect of KLF2-EVs on inhibiting Ly6C^high^ monocyte recruitment and ameliorating myocardial I/R injury. Finally, to exclude species differences, we used EVs derived from KLF2-overexpressing MCAECs to detect KLF2-EVs' effect and obtained similar results. Thus, our study provided a novel therapeutic approach and illustrated its clinical application for preventing I/R injury.

Spleen and bone marrow are the main sources of monocytes after I/R injury in the myocardium. In the acute period, splenic monocytes increase their motility, exit the spleen en masse, accumulate in the injured tissue, and participate in wound healing 22. Monocyte recruitment from the spleen reservoir usually peaks in 24 h after myocardial I/R injury and bone marrow gradually supplies monocyte to the infarct tissue 25. In this study, we observed that KLF2-EVs treatment reduced Ly6C^high^ Mo/Mø in the heart tissue and spleen reservoir on day 3 after myocardial I/R. This result indicated that the KLF2-EVs could not alter Ly6C^high^ monocytes recruited from the spleen reservoir. When KLF2-EVs inhibited Ly6C^high^ monocyte recruitment from BM, the Ly6C^high^ monocytes from the spleen gathered into infarct tissue, contributing to a decrease of Ly6C^high^ monocytes in the spleen reservoir. Also, no significant difference in peripheral blood Ly6C^high^ monocytes in the KLF2-EVs group could be compared to the I/R group on day 3 after myocardial I/R. But Ly6C^high^ Mo/Mø in the heart and blood Ly6C^high^ monocytes decreased after splenectomy. These data indicated that the spleen reservoir partly supplies Ly6C^high^ monocytes throughout acute inflammation 25, and plays a critical role in lessening Ly6C^high^ monocyte alternation in peripheral blood. The repressing effect on the spleen reservoir also resulted in the loss of BM monocyte kinetic change after KLF2-EVs administration. After splenectomy, retention of Ly6C^high^ monocytes in peripheral blood was reduced, and an increase in Ly6C^high^ monocytes in BM with KLF2-EVs treatment was evident. On the other hand, the MCP-1/CCR2 axis also facilitated the transfer of Ly6C^high^ monocytes from peripheral blood to tissues 22. Hence, when we applied KLF2-EVs to alter CCR2 expression, not only medullary Ly6C^high^ monocytes but also total extramedullary Ly6C^high^ Mo/Mø were altered. The infiltration of extramedullary Ly6C^high^ monocytes into infarct tissue was suppressed, contributing to the inconsistent results of Ly6C^high^ Mo/Mø in heart tissue and peripheral blood.

The I/R process after STEMI results from a highly complex and orchestrated series of events, including inflammatory and reparative phases. In the first 3-4 days, intense sterile inflammation and immune cell infiltration are initiated to digest and clear damaged cells. Then, cardiac repair begins with inflammation resolution, fibroblast proliferation, neovascularization, and scar formation that continue for several days 30, 31. Early inflammatory activation is necessary, but an appropriate and timely transition to later reparative and proliferative programs determines the quality of wound healing 32, 33. A disproportionately prolonged and high-intensity inflammatory phase after myocardial I/R injury is associated with worse clinical outcomes. Therefore, therapeutic modulation of the inflammatory phase holds promise for myocardium injury alleviation and post-infarction heart failure.

Nonselective inhibition of inflammation, such as corticosteroids and nonsteroidal anti-inflammatory drugs 8, selective targeting of pro-inflammatory signals, such as CD11/CD18 integrin inhibition 34, and P-selectin inhibition 35, after MI might have adverse effects on scar formation and myocardial remodeling, thereby raising the risk of cardiac rupture. Recently, new therapies emerged for regulating inflammation. For example, the canakinumab, which inhibits the IL-1β innate immunity pathway, significantly reduced the rate of recurrent cardiovascular events, but had a lower heart-targeting effect 36; theranostic nucleic acid-binding nanoprobes were shown to exert anti-inflammatory and cytoprotective effects in ischemic injury 37; platelet-targeted delivery of peripheral blood mononuclear cells to the ischemic heart restored cardiac function after I/R injury 38. Clearly, this field is in its infancy, and a concerted effort is needed to develop effective therapies to modify inflammation rationally.

In adult mammals, endothelial cells are the most widespread and abundant anti-inflammatory cells 39 that quickly transform towards the pro-inflammatory phenotype under pathological situations. Previous studies have shown that KLF2 was limited to endothelial cells within the vessel wall 12 and was a key transcriptional regulator to maintain endothelial physiological phenotype by inhibiting cytokine-mediated induction of E-selectin expression and vascular cell adhesion molecule (VCAM)-1 23. In the normal culture system, the ECs lose laminar flow stimulation and show phenotype alternation. We observed that HUVECs expressed a relatively high level of pro-inflammatory cytokines IL-1β and IL-6. Hence, we overexpressed KLF2 to mimic the physiological and anti-inflammatory phenotype in cultured HUVECs. We found that the upregulation of KLF2 promoted expression of anti-inflammatory cytokines and inhibited that of pro-inflammatory cytokines.

The small EVs have been shown to alter gene transcription and translation in recipient cells via delivering non-coding RNAs, including miRNAs and long non-coding RNAs (lncRNAs) 40. Several studies have illustrated beneficial effects of endothelial cell EVs *in vitro* and in animal models of sepsis, atherosclerosis, ischemia, and acute kidney injury 16, 41-43. For example, a localized injection of miRNA-21-enriched EVs effectively restored cardiac function after myocardial infarction 44. Similarly, hypoxia-elicited mesenchymal stem cell-derived exosomes facilitated cardiac repair through miR-125b-mediated prevention of cell death in myocardial infarction 45. In addition, Akbar et al endothelium-derived EVs promote splenic monocyte mobilization in myocardial infarction via transforming miRNA-126-3p and -5p 46. Our work firstly demonstrated that EVs derived from KLF2-HUVECs or KLF2-MCAECs could diminish infarct size and improve cardiac function post-myocardial I/R through inhibiting Ly6C^high^ monocytes recruitment from bone marrow, and miR-24-3p was further identified as an effective constituent in EVs. These results enlighten us EVs from differently modified ECs have different effect on the same disease model, and KLF2 was the crucial molecular in EVs' biological function.

We detected its constitutive expression in peripheral blood, heart tissue, and spleen was decreased in myocardial I/R mice compared with normal mice that might be related to enhancing Ly6C^high^ monocyte recruitment. However, miR-24-3p increased significantly in BM post-myocardial I/R and was considered a compensatory mechanism to maintain internal inflammation stability. Previous studies had reported miR-24 as one of the crucial regulators of cardiovascular pathology and inflammatory diseases. For example, miR-24 down-regulated cytokine synthesis in macrophages and inhibited vascular inflammation and abdominal aortic aneurysm pathology by targeting chitinase 3-like 1 27. miR-24-3p could attenuate myocardial I/R injury in mice by various mechanisms, such as suppressing RIPK1 expression 28, and regulate Keap1-Nrf2 pathway to reduce apoptosis 29. Nevertheless, miR-24 could also promote renal ischemic injury by stimulating apoptosis in endothelial and tubular epithelial cells, and miR-24 inhibition was protective 47. Thus, miR-24 might have diverse functions in different tissues and diseases.

In our study, we confirmed that miR-24-3p in KLF2-EVs restrained Ly6C^high^ monocyte recruitment by inhibiting CCR2 expression. We used i.v. route rather than the intramyocardial injection of KLF2-EVs, indicating that the dose of KLF2-EVs acting directly on myocyte was limited. Whether KLF2-EVs could affect myocytes via Keap1- Nrf2 or RIPK1 pathway should be explored in the future.

For monocyte recruitment, the mainstream viewpoint holds that in 24 hours after coronary ligation, resident heart macrophages (CD11b+ F4/80+ Ly6C^low^) are completely lost while inflammatory macrophages (CD11b+ F4/80- Ly6C^high^) rise sharply, and on day 4 the reverse situation occurs by the preferential accumulation of Ly6C^low^ monocytes 48. This biphasic monocyte recruitment pattern is orchestrated by increased MCP-1 expression in infarcts in the first phase, attracting Ly6C^high^ CCR2^high^ monocytes, and increased fractalkine expression in the second phase, favoring Ly6C^low^ CX3CR1^high^ monocytes that arise from Ly6C^high^ CCR2^high^ monocytes 3. Generally, Ly6C^high^ monocytes are typically regarded as the central inflammatory culprits 49, and infarct macrophages are derived from recruited cells in the peripheral blood until two weeks post-ischemia injury 48.

The functional characterization of monocyte subtypes in cardiovascular diseases has attracted therapeutic exploration in monocyte recruitment. The interest had mostly focused on targeting the accumulation of Ly6C^high^ monocytes, especially blocking the MCP-1/CCR2 axis. However, these therapeutic strategies failed to treat vascular diseases in animal models or clinical trials. Here we discovered that exosomes from KLF2 over-expressing ECs could prevent myocardial I/R injury via altering Ly6C^high^ monocyte recruitment. As opposed to the myocardium local injection or intracoronary injection, the EVs were injected intravenously to regulate systemic inflammation, which could be easily translated to the treatment of clinical disorders. We also reestablished anti-inflammatory phenotype of the endothelium via KLF2-EVs in the I/R model, not perturbing the inheritance background. Hence this study suggested the therapeutic potential of KLF2-EVs for clinical application to prevent I/R injury.

There are some limitations to our study. Although miR24-3p antagomir reversed KLF2-EVs effect, specific mechanisms should be investigated in CCR2^-/-^ mice and bone marrow transplantation. Also, endothelial cells can exert important actions through exosome-independent effects, and KLF2 is known to control a considerable fraction of the endothelial cell transcriptome 50. In the future, we plan to examine the broad effects of endothelial KLF2 overexpression in the I/R heart. In addition, the predominate site of clearance for EVs is the spleen and liver, and we have to inject large amount EVs to confirm enough EVs to exert its effect. Intramyocardial injection might need less EVs but it's difficult to carry out in clinical treatment. Therefore, we begin a new study to modify EVs by CD47, which could help EVs escape from macrophage phagocytosis and degradation in peripheral blood. We hope this 2^nd^ generation KLF2-CD47-EVs could exert its protective effect at a low dosage and be used in clinical application for STEMI patients.

## Conclusions

Our study demonstrated that KLF2-transduced ECs-derived EVs attenuated myocardial I/R injury by inhibiting Ly6C^high^ monocyte recruitment from bone marrow. We also provided evidence that miR-24-3p packaged in EVs was involved in modulating Ly6C^high^ monocyte recruitment by targeting CCR2. This study shed new light on the application of KLF2-EVs as a potential therapeutic agent for myocardial I/R injury.

## Supplementary Material

Supplementary figures and tables.

## AbbrEViations

STEMI: ST-segment elevation myocardial infarction
KLF2: Krüppel-Like Factor 2
ECs: endothelial cells
EVs: extracellular vesicles
KLF2-EVs: extracellular vesicles from KLF2-overexpressed endothelial cells
miR-24-3p: miRNA-24-3p
I/R: ischemia/reperfusion
PCI: percutaneous coronary intervention
MCP-1: monocyte chemoattractant protein-1
CCR2: C-C chemokine receptor type 2
CX3CR1: CX3C chemokine receptor 1
Mo/Mø: monocytes/macrophages
HUVECs: human umbilical vein endothelial cells
MCAECs: mouse coronary endothelial cells
PBS: phosphate-buffered saline
LAD: left anterior descending
KLF2-HUVECs: transduced HUVECs with a lentivirus vector encoding KLF2
vector-HUVECs: transduced HUVECs with an empty vector
KLF2-EVs: EVs derived from KLF2-ECs
vector-EVs: EVs derived from vector-ECs
NTA: nanoparticle tracking analysis
TEM: transmission electron microscopy
EF: ejection fraction
FS: fractional shortening
UCG: Echocardiography
LVDd: left ventricular end-diastolic diameter
Cl_2_MDP: dichloromethylene diphosphonate
LPS: lipopolysaccharide
NC: negative control
