# Supplementary material for: Extracellular vesicles derived from Krüppel-Like Factor 2-overexpressing endothelial cells attenuate myocardial ischemia-reperfusion injury by preventing Ly6Chigh monocyte recruitment
Source: Theranostics. 2020 Sep 18;10(25):11562–79. doi: 10.7150/thno.45459 (PMC7545985; doi:10.7150/thno.45459)
Supplement: Supplementary file 1 — Supplementary figures and tables. [file thnov10p11562s1.pdf]

Supplementary Materials

Supplementary Methods

Cell experiments protocol

The human umbilical vein endothelial cells (HUVECs) were cultured in endothelial cells (ECs) culture medium (Science Cell, 1001) supplemented with 10% fetal bovine serum (FBS, Science Cell, 0025), 1% endothelial cell growth supplement (Science Cell, 1052) and 1% Penicillin/Streptomycin (Science Cell, 0503). The cultures were maintained at 37 °C in 5 % CO2 and 95 % humidity. Culture medium was changed every 3 days. HUVECs between passage 3 to 6 were used for the subsequent experiments.

In order to simulate laminar flow circumstance of vessel, in according to previous study described[13], HUVECs were inoculated on fibronectin-coated Thermanox coverslips (NUNC, Napierville, IL) in ECs culture medium. 24 hours later, CellMax Quad positive-displacement pump (Cellco, Germantown, MD) was applied in a parallel plate-type flow chamber with steady flow (25 dyne/cm2) to create shear stress.

The mouse coronary endothelial cells (MCAECs) were isolated from mouse coronary endothelium according to the protocol and video. C57BL/6 mice were purchased (4 weeks of age) from the Model Animal Research Center of Nanjing University, and were injected intraperitoneally with 0.1 ml of Heparin to prevent blood coagulation and then were anesthetized by 1.5% isoflurane inhalation. Remove carefully heart and put into in a beaker with 1× Kreb's. The heart tissue was digested with enzyme solution and conjugated with magnetic beads that took rat anti-mouse CD31 antibody. MCAECs were gained through magnetic activated cell sorting. MCAECs purity test was performed by staining cells with an endothelial cell surface marker CD31 and Dil-acLDL. The percentage of positive cells which exhibited color was over 90% in MCAECs. Such a result indicated that the technique was performed successfully.

Murine macrophage RAW264.7 cells were bought from the American Type Culture Collection and cultured in DMEM with high glucose supplemented with 10% FBS and 1% Penicillin/Streptomycin. The cultures were maintained at 37 °C in 5 % CO2 and 95 % humidity.

The primary BM-derived macrophages (BMDMs) was extracted from C57BL/6 mice (4 weeks of age). First, mice were suspended in alcohol and bacterial femora were separated into icy PBS. Next, both joints were cut in clean bench and RPMI 1640 medium was injected into BM cavity for cells lavage. Collected cells were washed with PBS and cultured with RPMI 1640 medium supplemented with 10% FBS, 1% Penicillin/Streptomycin and 20ng/ml M-CSF for seven days. Culture medium was changed every 3 days. The adhesive cells were BMDMs.

Recombinant lentivirus vector assembly and transduction into ECs

In the present study, we first constructed a GV358 vector (Ubi-MCS-3FLAG-SV40-EGFP-IRES-puromycin) carrying Krüppel-Like Factor 2 (KLF2) cDNA and was co-transfected with lentivirus backbone plasmid into HEK293A cells to produce the recombinant lentivirus vector Lv-KLF2. Another GV358 vector without KLF2 cDNA was used to generate empty viruses as controls (empty vector). ECs were cultured at a density of 1×10^6 cell/ml in six-well plates overnight. The lentiviruses (1.5×10^9 TU/ml) were diluted with 1ml of complete medium containing HitransG P (1 μg/ml, GeneChem, China) and then added to ECs. After transfection for 12 hours at 37 °C, the medium was changed to fresh virus-free medium. Continually culturing for 72 hours, we could detect successfully transfected cells presented green fluorescence (GFP positive) with a fluorescence microscopy (IX 53, Olympus Corporation, Japan). Next, the puromycin (5 μg/ml) was applied to the culture medium to remove negative cells, and the selected cells were KLF2-tranfected ECs. The KLF2 expression levels were measured by quantitative reverse transcriptase polymerase chain reaction (qRT-PCR) and western blot.

Labelling of exosomes with PKH67

To observe the internalization of exosome into monocytes, extracellular vesicles (EVs) were labeled with PKH67 dye (Sigma) according to the manufacturer's instructions and washed with PBS followed by centrifugation at 100,000 g for 1 hour at 4 °C. For vivo experiment, the PKH67-labeled KLF2-EVs (3μg/g) was injected intravenously into I/R injury mice. After 24 hours, BM cells lavage was performed for flow cytometry analysis. For vitro experiment, the PKH67-labeled KLF2-EVs (30 μg/ml) was co-cultured with RAW264.7 cells. After 12 hours, the cells were washed with PBS and stained with DAPI (Ribobio). Finally, the cells were photographed with a confocal microscope (Olympus FV1200).

Evan’s blue and 2,3,5-Triphenyltetrazolium Chloride (Evans blue/TTC) staining

Briefly, 3 days following ischemia/reperfusion (I/R) injury, mice were anesthetized and the left anterior descending (LAD) coronary artery was re-occluded at the previous ligation and aorta was ligated at root, and then 0.1 mL of 1% Evans blue (Sigma-Aldrich, St. Louis, MO, USA) was injected into the left ventricular (LV) cavity. The heart was quickly excised, washed with normal saline and immediately frozen. Afterward, the heart tissue was cut in short-axis direction at 1mm thickness and were incubated at 37°C in 1.5 % 2,3,5-triphenyltetrazolium chloride (TTC; Sigma-Aldrich) for 15 minutes. The heart pieces were unfolded in glass slide and digitally photographed. LV area, areas at risk (AAR), and infarct size (IS) were determined by computerized planimetry and comprehensively analyzed in serial sections of each mice using Image J software (version 1.38, National Institutes of Health, Bethesda, MD, USA).

Histology

Histology of hearts was assessed on day 3 after myocardial I/R. Hearts were arrested in diastole late term with intraventricular injection of 10% potassium chloride (KCl). The heart was quickly excised, washed with normal saline and were fixed with 4% phosphate-buffered formalin. After gradually dehydration and embedded in paraffin, the tissue was cut into transverse sections at 5μm thickness.

For H.E. staining, tissue slices were stained with hematoxylin and eosin to analyze the global heart morphology and inflammatory cell infiltration. For inflammatory cell infiltration analysis, we randomly selected ten fields (400× magnification) within the infarct zone of each heart. The percentage of inflammatory cells was evaluated by counting the number of inflammatory cells cellular nuclei and compared with the total number of cellular nuclei in the same view of field.

For Masson trichrome staining, tissue slices were stained with Masson trichrome or Sirius red according to the manufacturer’s instruction. To assess scar area, we digitally photographed the heart tissue slides and calculated the ratio of the collagen (blue-stained) area to the total tissue area. The collagen-rich border zone of the vessels was excluded from the analysis.

For immunofluorescence staining, the deparaffinized tissue slides were boiled in citrate buffer at 100 °C for one hour for antigen retrieval and then were blocked in 1 % FBS at room temperature for one hour followed by overnight incubation with primary antibodies at 4 °C. To determine $Ly6C_{high}$ and $Ly6C_{low}$ macrophages distribution, we used a double staining with FITC–conjugated F4/80 antibody (Invitrogen, USA) and PE–conjugated inducible Nitric Oxide Synthase (iNOS) or CD163 antibody (Invitrogen, USA) in conjunction with DAPI. Images were captured and processed using a fluorescence microscopy (IX 53, Olympus Corporation, Japan). Count and calculate separately F4/80+iNOS+ cells ratio and F4/80+CD163+ cells ratio in three fields (400× magnification) from five animals with each treatment.

Echocardiography

Cardiac function was evaluated professional technicians using Color Doppler Echocardiography (VEVO 1100 Imaging system; VisualSonics Inc). Mice were under light anesthesia and two-dimensional short and long axes imaging were acquired to calculate LV functional parameters. LV end-systolic diameter (LVID; d), LV end-diastolic diameter (LVID; s), interventricular septal thickness (IVS) and LV posterior wall thickness (LVPW) (end-diastolic and end-systolic) were measured from at least three consecutive cardiac cycles on the M-mode tracings. LV fractional shortening (FS %) was determined as [(LVID;d–LVID; s)/LVID; d]× 100. LV ejection fraction (EF) was calculated as: EF (%) = ((LV Vol; d-LV Vol; s)/LV Vol; d) × 100. LV Vol; d = ((7.0 / (2.4 + LVID; d)) × $LVID;d^3$); LV Vol; s = ((7.0 / (2.4 + LVID;s)) × $LVID;s^3$).

Migration

Migration experiments were performed through Transwell experiment and cell scratch wound healing assay. For Transwell experiment, RAW264.7 cells/BMDMs were resuspended in RPMI 1640 medium containing 10% FBS and 5×10^4 cells were seeded on a gelatin-coated polycarbonate membrane with 8-mm pores. Migration was induced by the addition of monocyte chemoattractant protein-1 (MCP-1, 10ng/ml, Invitrogen, USA). After 6 hours, non-migrating cells were removed and the polycarbonate membranes were fixed and stained with Giemsa dye. The number of migrated cells was calculated in 200× high-power field. Experiments were repeated at least three times. For scratch wound healing assay, RAW264.7

cells/BMDMs were seeded in six-well plates and culture until confluent. Using a yellow pipette tip make a straight scratch in one direction, and gently wash the well twice with medium to remove the detached cells. Culture cells for another 48 hours and fix the cells with 4% paraformaldehyde for 30 min. Finally, take at least three photos of peer well on a microscope and measure length using software.

Quantitative reverse transcriptase polymerase chain reaction (qRT-PCR)

Total RNA was extracted from frozen or fresh tissues and cells with TRIzol® Reagent (Invitrogen) and reversely transcribed into cDNA with the Transcriptor First Strand cDNA Synthesis Kit (Roche LifeScience) for mRNA and miRNA. The expression levels of genes were quantified with the SYBR Green Reagents Kit (Roche LifeScience), and was normalized to that of the internal reference gene GAPDH or U6 to calculate the $2^{-\Delta Ct}$ value. Data were showed as the mean ± SD from at least three repeated experiments. The primers used are listed in Table S9.

Western blot

The total protein was extracted from tissue or cells and boiled with loading buffer for 5 minutes. Protein electrophoresis was conducted using TGX FastCast acrylamide Kit (BIO RAD Laboratories Inc.), and then transferred to PVDF membrane (Millipore, Bedford, MA, USA). After blocking in 5% dry milk, the membrane was incubated with primary antibodies (1:1000 or 1:500). Next, the membrane was washed in Tris-buffered saline with Tween 20 (0.1%) (TBS-T) for least three times and was incubated with horseradish peroxidase-conjugated secondary antibodies (1:10000). Detection was performed using ECL Prime (GE Healthcare, Logan, UT, USA) according to the manufacturer’s instructions. The antibodies used are listed in Table S10.

Flow cytometry analysis

For tissue cytometry analysis, we harvested the whole heart and cut infarct tissue from left ventricle. Then single-cell suspensions of tissues were obtained using gentle MACS™ Dissociator (Miltenyi Biotec). Samples were resuspended in staining buffer (R&D Systems) and stained with CD11b-FITC (BD Bioscience) and Ly6C-PE (eBioscience) for lucifugal incubation 30 minutes at 4°C. Flow cytometry was performed with FACS Aria flow

cytometer (BD Bioscience), and data were analyzed with FlowJo software (TreeStar, Ashland, OR). For flow cytometry data analysis, we used percentage to evaluate Ly6C$_{high}$ and Ly6C$_{low}$ monocytes/macrophages. For example, in heart tissue, percentage represented Ly6C$_{high}$ or Ly6C$_{low}$ macrophages number divided by all heart viable cells number, and in peripheral blood, percentage represented Ly6C$_{high}$ or Ly6C$_{low}$ monocytes number divided by all blood viable cells number. For FACS gating strategy, we combined fluorescence and intrinsic properties at the same time. First plot gating according to FSC forward scatter for live cells, and then use CD11b and Ly6C to delineate monocyte phenotype subsets. The boundaries between "positive" and "negative" staining cell populations were defined with isotype control.

Enzyme-linked immunosorbent assay (ELISA)

Blood was drawn from mice under anesthesia by removing eyeball with a syringe pre-loaded with 600U/ml heparin anticoagulant, and was centrifuged at 300g for 5 min at 4 °C for depart serum from cell. IL-1β, IL-10, CCL2 and CX3CL1 concentration was determined with specific ELISA Kit (MultiSciences), and normalized to the volume of blood samples.

Total RNA extraction and microRNA microarray

The total mRNA in EVs was extracted by using TRIzol® Reagent (Invitrogen) according to the manufacturer’s instructions, and RNA purity was checked with the NanoPhotometer® spectrophotometer (IMPLEN, CA, USA). RNA concentration was measured with Qubit® RNA Assay Kit in Qubit® 2.0 Flurometer (Life Technologies, CA, USA). RNA integrity was assessed with the RNA Nano 6000 Assay Kit of the Agilent Bioanalyzer 2100 system (Agilent Technologies, CA, USA). Microarray hybridization and analysis were performed using Illumina HiSeqTM2500/MiSeq, based on the miRBase database. The data output was received in Excel spreadsheets, and microRNA family analysis, GO enrichment analysis and Target gene prediction results were demonstrated in plots of Venn (Figure 7A), Volcano (Figure 7B), Heatmap (Figure S11A) and Bubble map (Figure S11B). Three duplicates of EVs from KLF2-EVs group and vector-EVs group were sequenced.

Prediction of target genes and Dual-luciferase reporter assay

The potential target genes of the miRNA24-3p (miR-24-3p) were predicted with TargetScan7.1 and mirdbv5. The sequences of miR-24-3p (UGGCUCAGUUCAGCAGGAACAG) between mouse and human were highly conserved. The dual-luciferase reporter was constructed with either wild-type or mutant promoter. The respective mRNA sequences of C-C chemokine receptor type 2 (CCR2) mRNA 3'-UTR segment (5'…GUCUUCCCUGAAUUGAGCCAAAAAUGUUCUUCCCU… 3') and its respective mutant segment (5'…GUCUUCCCUGAAUCAUUCUAAAAAUGUUCUUCC CU … 3') were amplified and corresponding cDNA segments were inserted into the pRL-TK vector (KeyGEN, China). Cultured HEK293 cells were transfected with these vectors as well as miR-24-3p mimic, miR-24-3p inhibitor or nonsense stem-loop using Lipofectamine (Invitrogen, USA). After 48 hours, we detected and analyzed the luciferase activity in the use of Dual-Luciferase® Reporter Assay System 10-Pack (Promega, USA). The relative activity of the reporter gene was compared among these groups to determine the target of microRNA. Three replicates were performed.

Transfection of miRNA mimics and inhibitors

RAW264.7 cells were transfected with the indicated miR24-3p mimics (50nM, Ribobio, miR24-3p inhibitor (100nM, Ribobio) companied with KLF2-EVs using Lipofectamine 3000 (Invitrogen) according to the manufacturer's instructions. We used scrambled pre-microRNA as a negative control (NC), and the effect of transfection was examined by qRT-PCR. Next, the cells were seeded to measure migration with MCP-1.

Figure S1

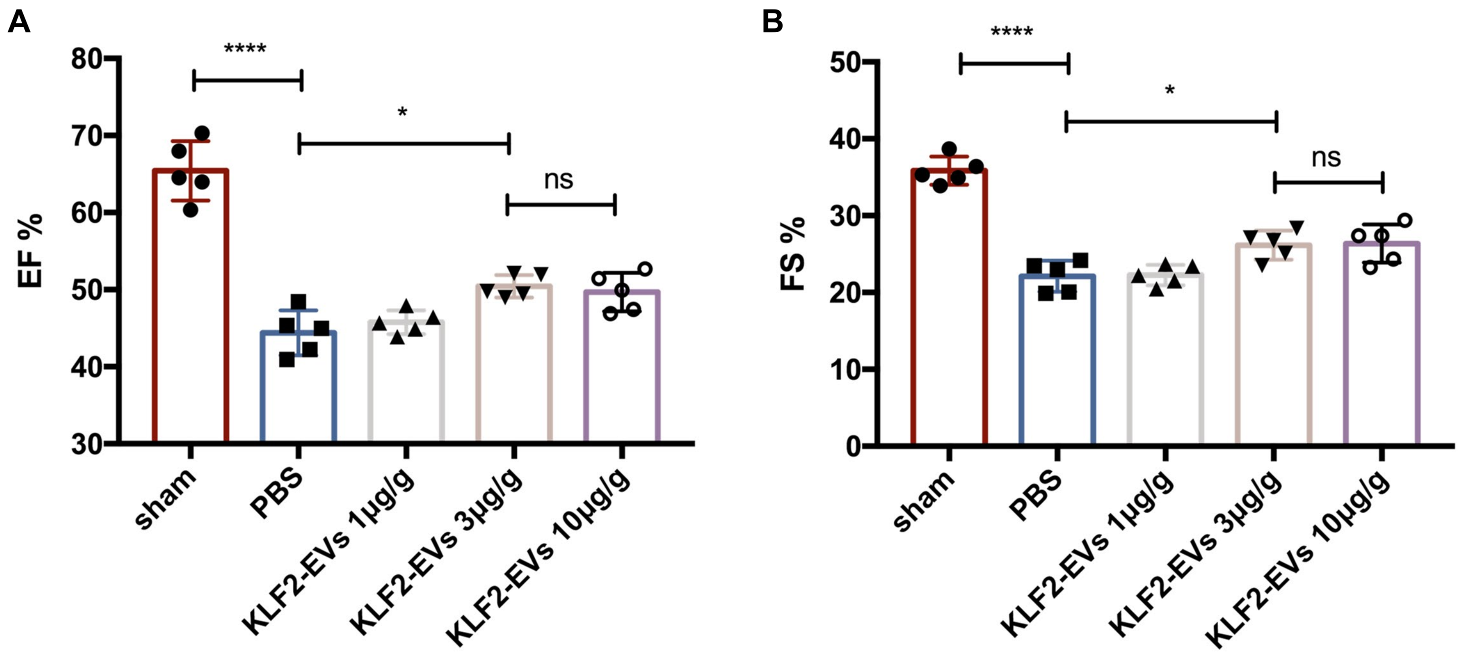

A
****
*
ns
80
70
60
50
40
30
EF %
sham
PBS
KLF2-EVs 1μg/g
KLF2-EVs 3μg/g
KLF2-EVs 10μg/g
B
****
*
ns
50
40
30
20
10
0
FS %
sham
PBS
KLF2-EVs 1μg/g
KLF2-EVs 3μg/g
KLF2-EVs 10μg/g

Figure S2

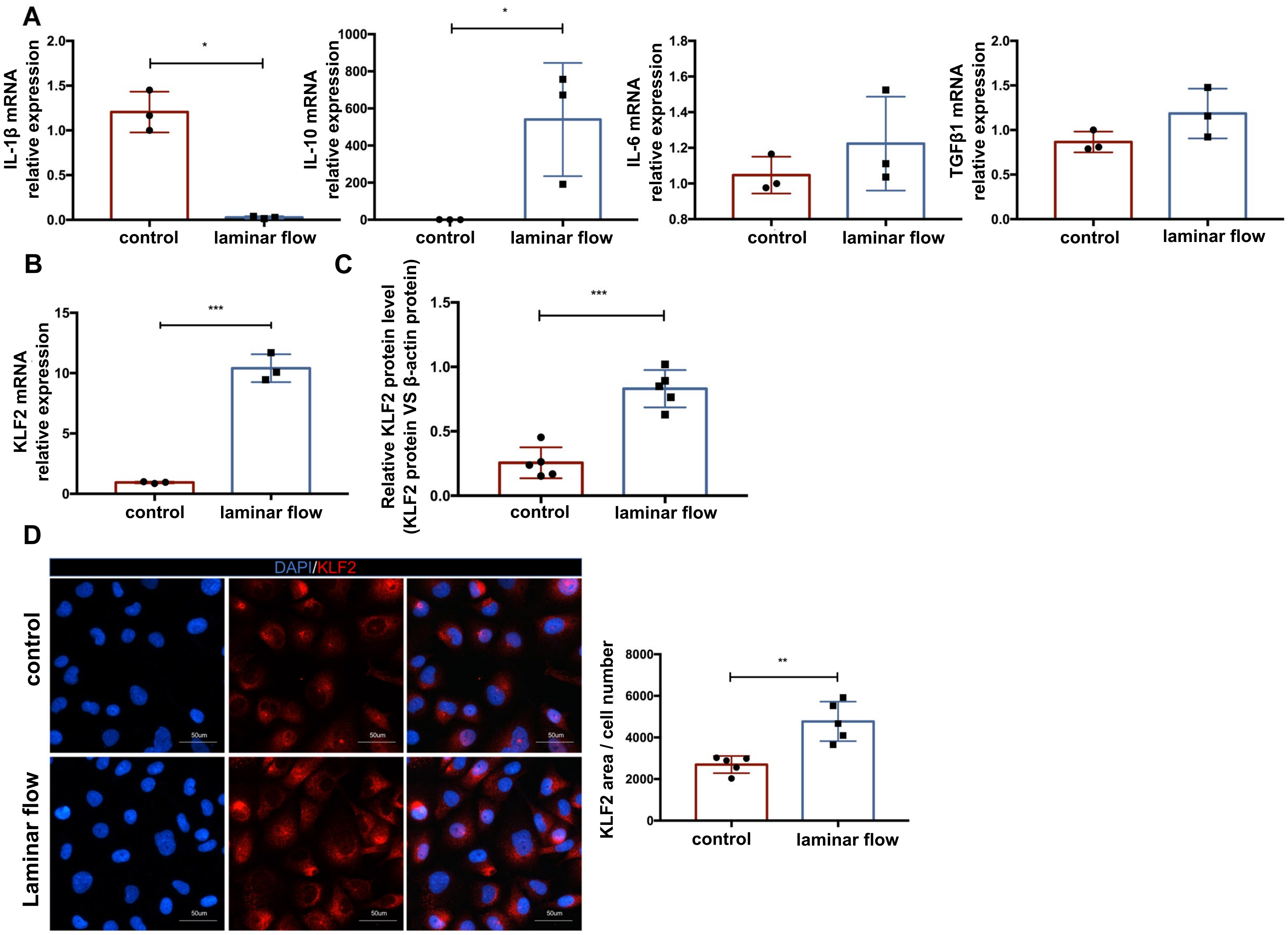
A
IL-1β mRNA relative expression
2.0
1.5
1.0
0.5
0.0
*
control
laminar flow
IL-10 mRNA relative expression
1000
800
600
400
200
0
*
control
laminar flow
IL-6 mRNA relative expression
1.8
1.6
1.4
1.2
1.0
0.8
control
laminar flow
TGFβ1 mRNA relative expression
2.0
1.5
1.0
0.5
0.0
control
laminar flow
B
KLF2 mRNA relative expression
15
10
5
0
***
control
laminar flow
C
Relative KLF2 protein level (KLF2 protein VS β-actin protein)
1.5
1.0
0.5
0.0
***
control
laminar flow
D
DAPI/KLF2
control
Laminar flow
50um
KLF2 area / cell number
8000
6000
4000
2000
0
**
control
laminar flow

Figure S3

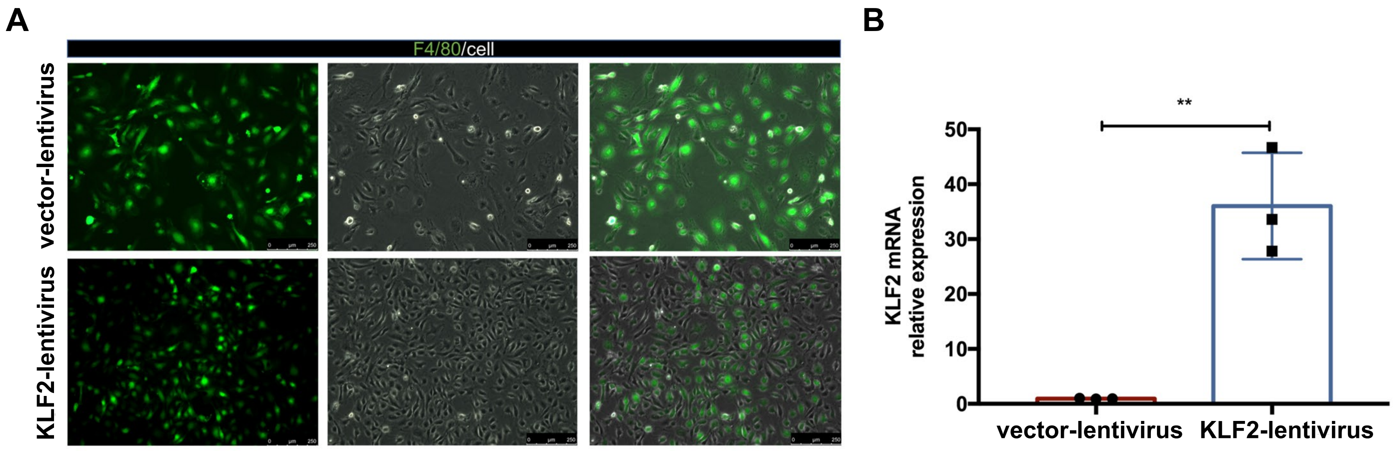
A
F4/80/cell
vector-lentivirus
KLF2-lentivirus
B
**
KLF2 mRNA
relative expression
50
40
30
20
10
0
vector-lentivirus
KLF2-lentivirus

Figure S4

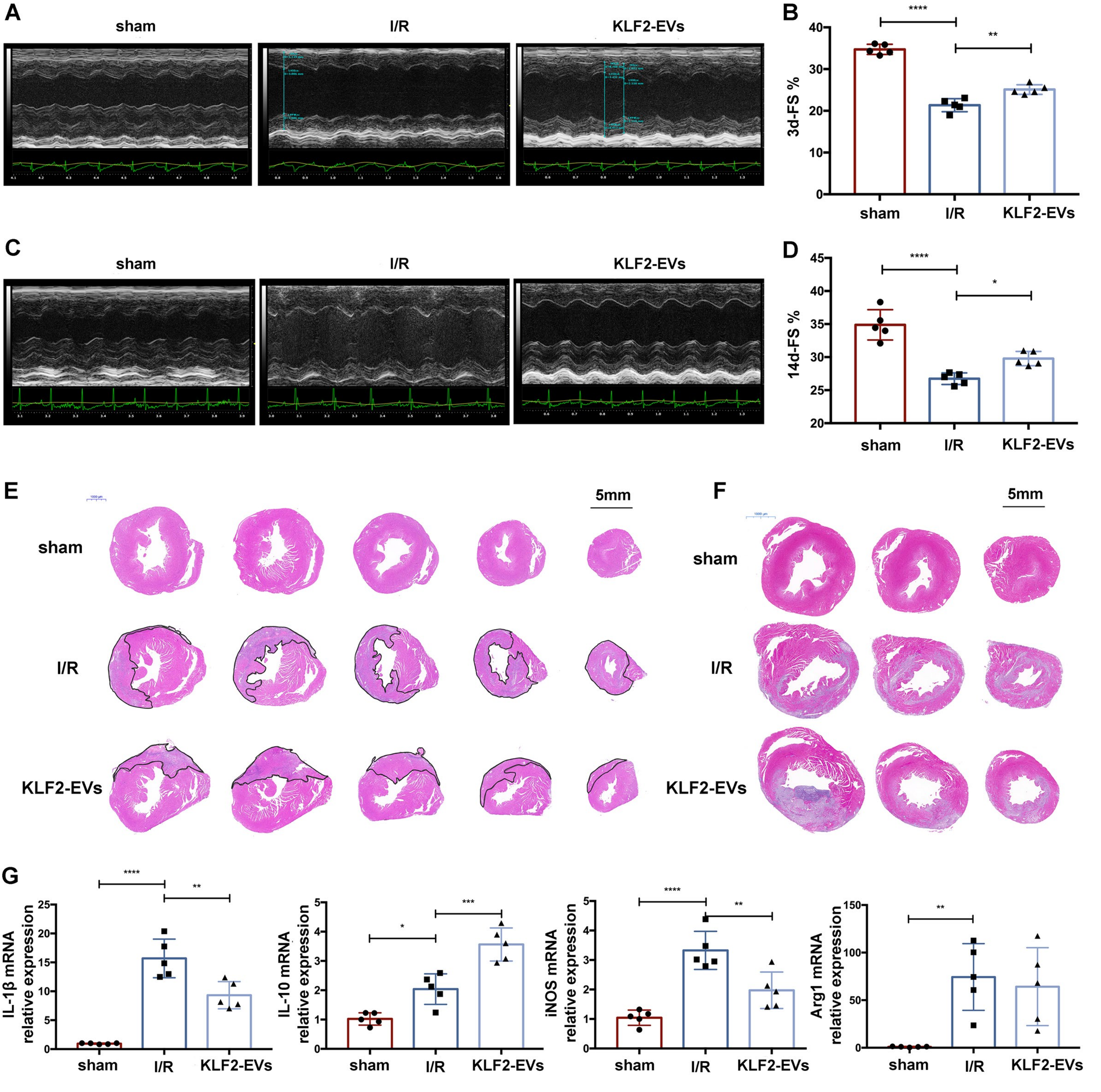

A
sham
I/R
KLF2-EVs
B
3d-FS %
sham
I/R
KLF2-EVs
C
sham
I/R
KLF2-EVs
D
14d-FS %
sham
I/R
KLF2-EVs
E
5mm
sham
I/R
KLF2-EVs
F
5mm
sham
I/R
KLF2-EVs
G
IL-1β mRNA relative expression
IL-10 mRNA relative expression
iNOS mRNA relative expression
Arg1 mRNA relative expression
sham
I/R
KLF2-EVs

Figure S5

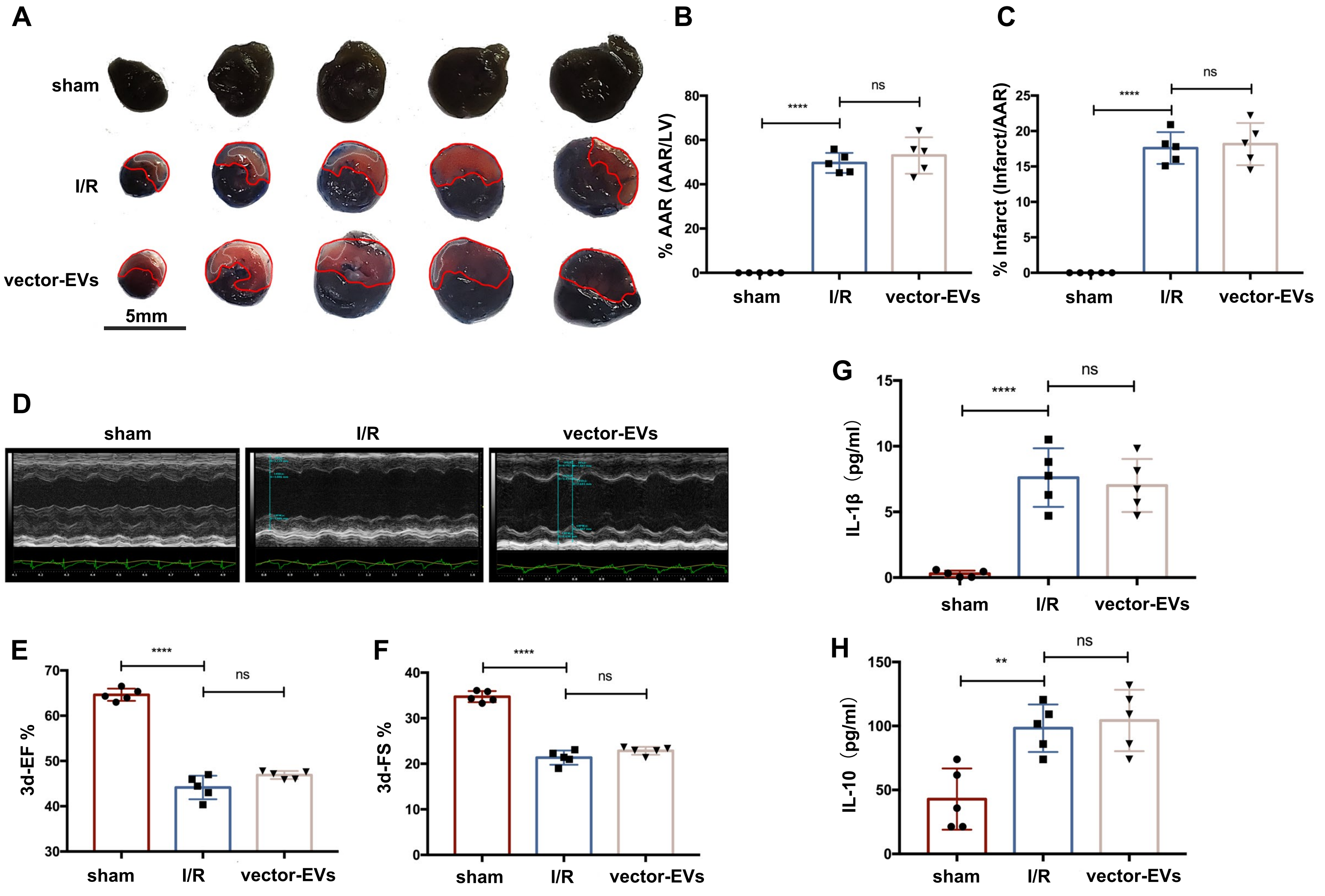
A
sham
I/R
vector-EVs
5mm
B
% AAR (AAR/LV)
****
ns
sham
I/R
vector-EVs
C
% Infarct (Infarct/AAR)
****
ns
sham
I/R
vector-EVs
D
sham
I/R
vector-EVs
E
3d-EF %
****
ns
sham
I/R
vector-EVs
F
3d-FS %
****
ns
sham
I/R
vector-EVs
G
IL-1β（pg/ml）
****
ns
sham
I/R
vector-EVs
H
IL-10（pg/ml）
**
ns
sham
I/R
vector-EVs

Figure S6

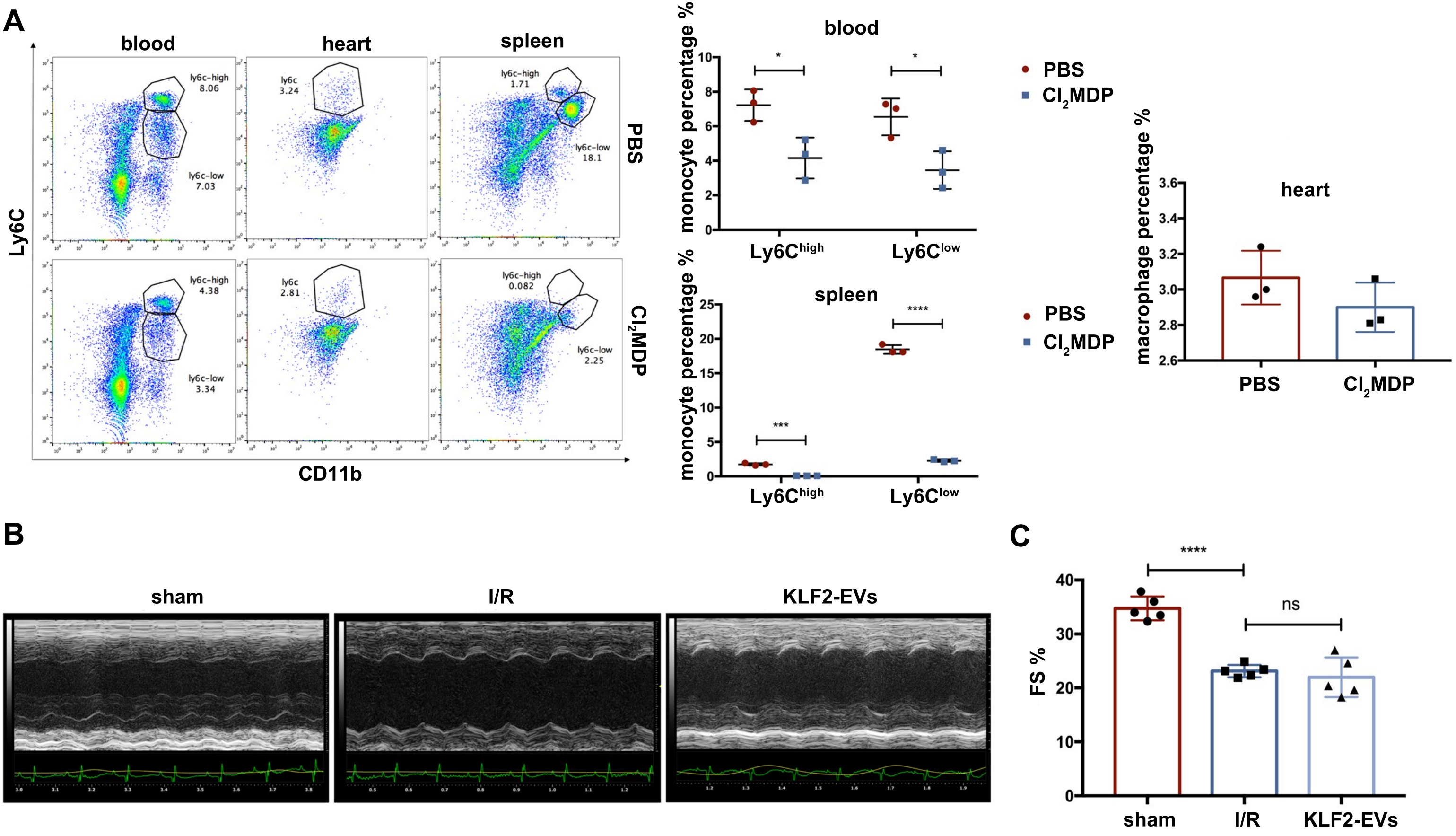
A
blood
heart
spleen
ly6c-high
8.06
ly6c-low
7.03
ly6c
3.24
ly6c-high
1.71
ly6c-low
18.1
PBS
ly6c-high
4.38
ly6c-low
3.34
ly6c
2.81
ly6c-high
0.082
ly6c-low
2.25
Cl2MDP
Ly6C
CD11b
monocyte percentage %
blood
Ly6Chigh
Ly6Clow
spleen
PBS
Cl2MDP
macrophage percentage %
heart
B
sham
I/R
KLF2-EVs
C
FS %
ns
sham
I/R
KLF2-EVs

Figure S7

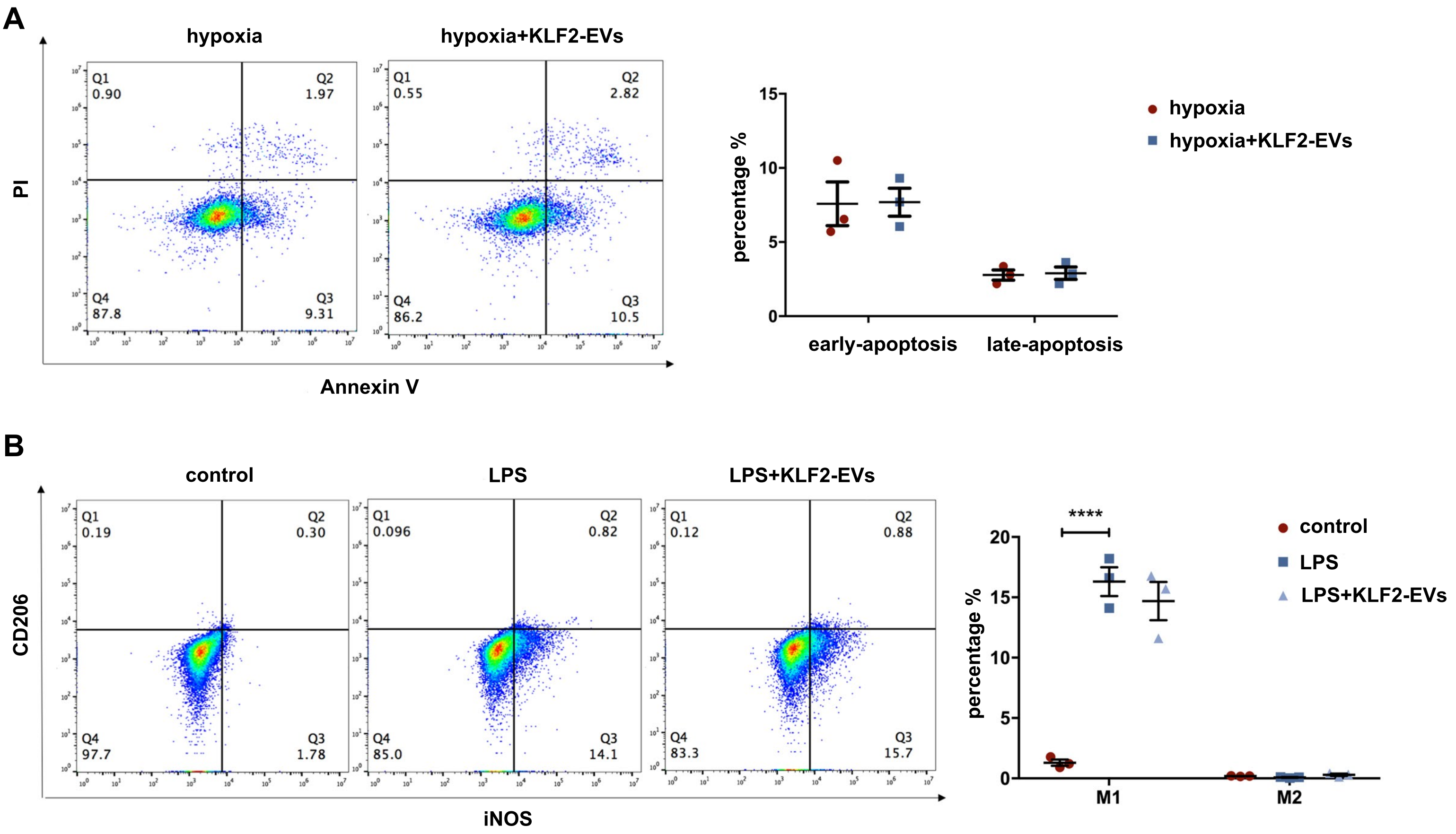

A
hypoxia
hypoxia+KLF2-EVs
PI
Annexin V
Q1
0.90
Q2
1.97
Q4
87.8
Q3
9.31
Q1
0.55
Q2
2.82
Q4
86.2
Q3
10.5
percentage %
15
10
5
0
early-apoptosis
late-apoptosis
hypoxia
hypoxia+KLF2-EVs
B
control
LPS
LPS+KLF2-EVs
CD206
iNOS
Q1
0.19
Q2
0.30
Q4
97.7
Q3
1.78
Q1
0.096
Q2
0.82
Q4
85.0
Q3
14.1
Q1
0.12
Q2
0.88
Q4
83.3
Q3
15.7
****
percentage %
20
15
10
5
0
M1
M2
control
LPS
LPS+KLF2-EVs

Figure S8

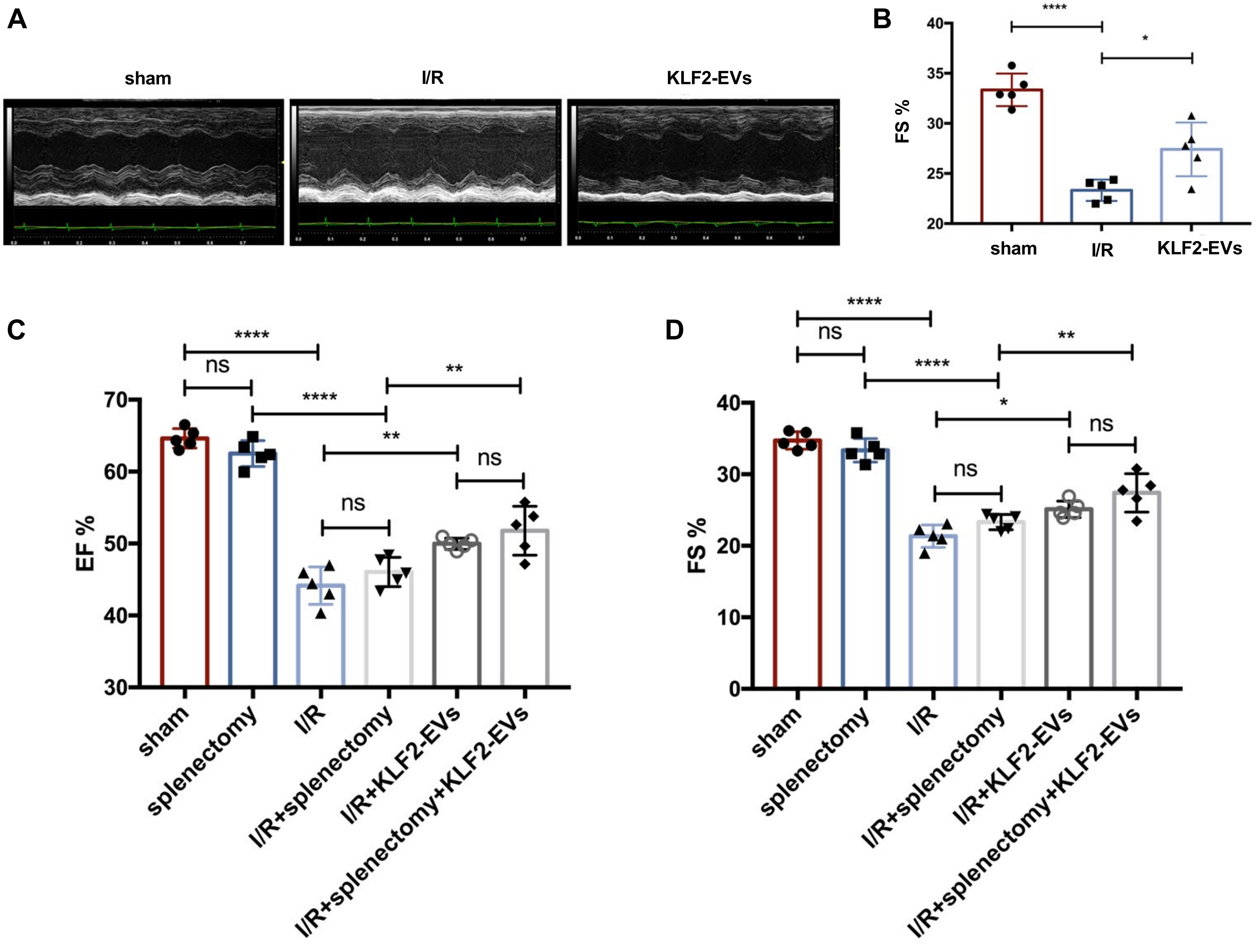
A
sham
I/R
KLF2-EVs
B
FS %
sham
I/R
KLF2-EVs
C
EF %
sham
splenectomy
I/R
I/R+splenectomy
I/R+KLF2-EVs
I/R+splenectomy+KLF2-EVs
D
FS %
sham
splenectomy
I/R
I/R+splenectomy
I/R+KLF2-EVs
I/R+splenectomy+KLF2-EVs

Figure S9

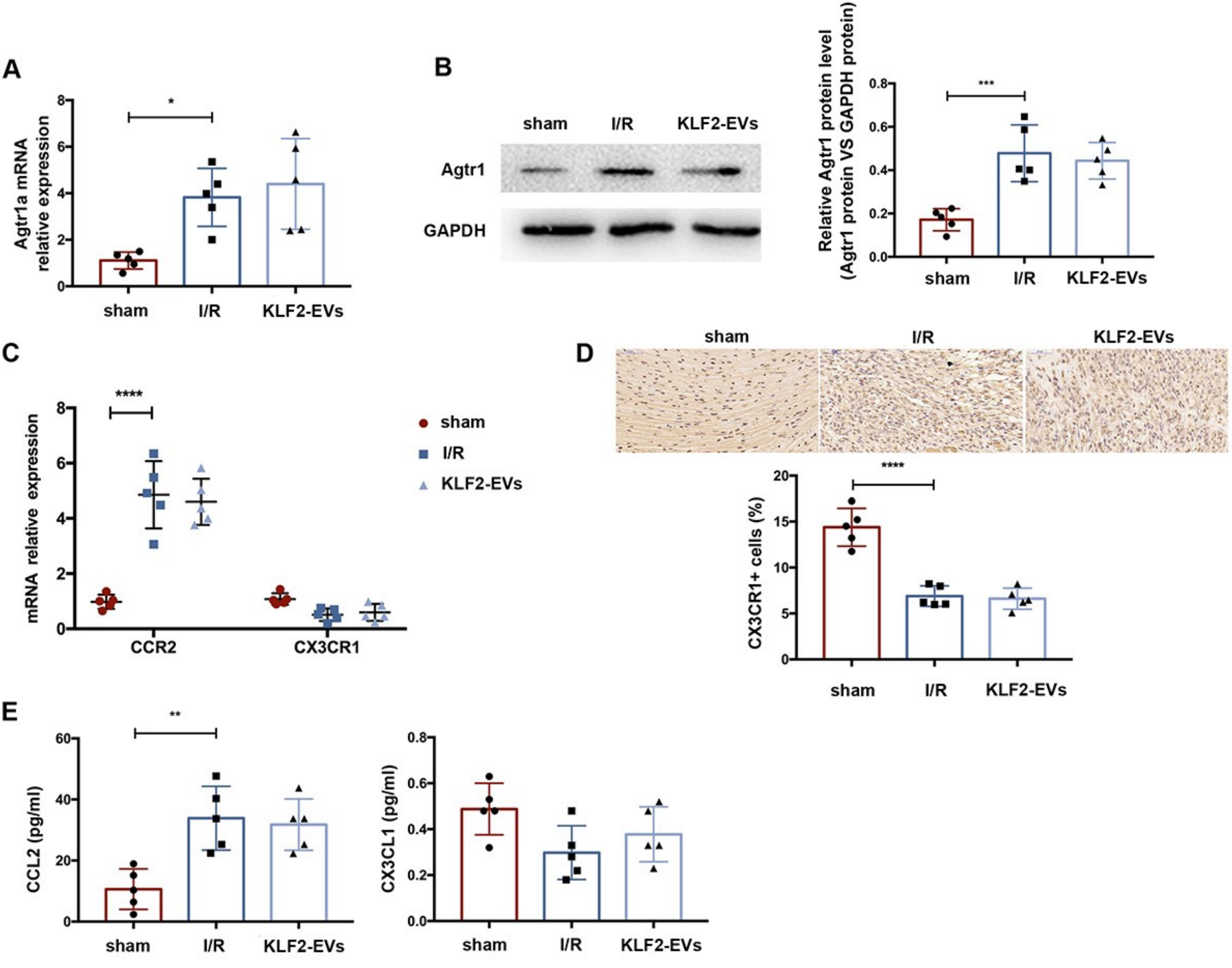

A
Agtr1a mRNA relative expression
*
sham
I/R
KLF2-EVs
B
sham
I/R
KLF2-EVs
Agtr1
GAPDH
Relative Agtr1 protein level (Agtr1 protein VS GAPDH protein)
***
C
mRNA relative expression
****
CCR2
CX3CR1
D
CX3CR1+ cells (%)
E
**
CCL2 (pg/ml)
CX3CL1 (pg/ml)

Figure S10

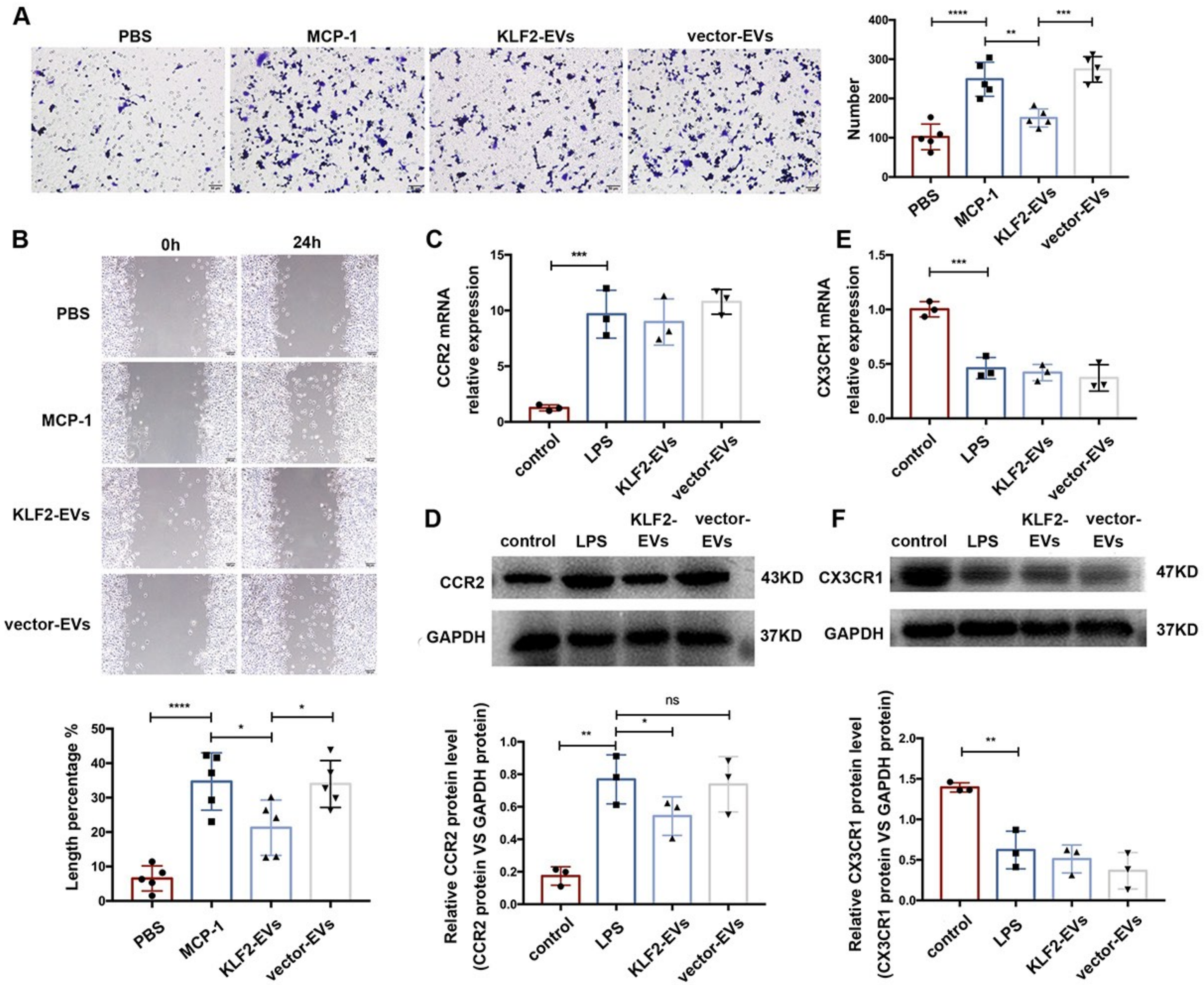
A
PBS
MCP-1
KLF2-EVs
vector-EVs
Number
****
**
***
B
0h
24h
PBS
MCP-1
KLF2-EVs
vector-EVs
Length percentage %
****
*
*
C
CCR2 mRNA relative expression
***
control
LPS
KLF2-EVs
vector-EVs
E
CX3CR1 mRNA relative expression
***
D
control
LPS
KLF2-EVs
vector-EVs
CCR2
43KD
GAPDH
37KD
Relative CCR2 protein level (CCR2 protein VS GAPDH protein)
**
*
ns
F
CX3CR1
47KD
GAPDH
37KD
Relative CX3CR1 protein level (CX3CR1 protein VS GAPDH protein)
**

Figure S11

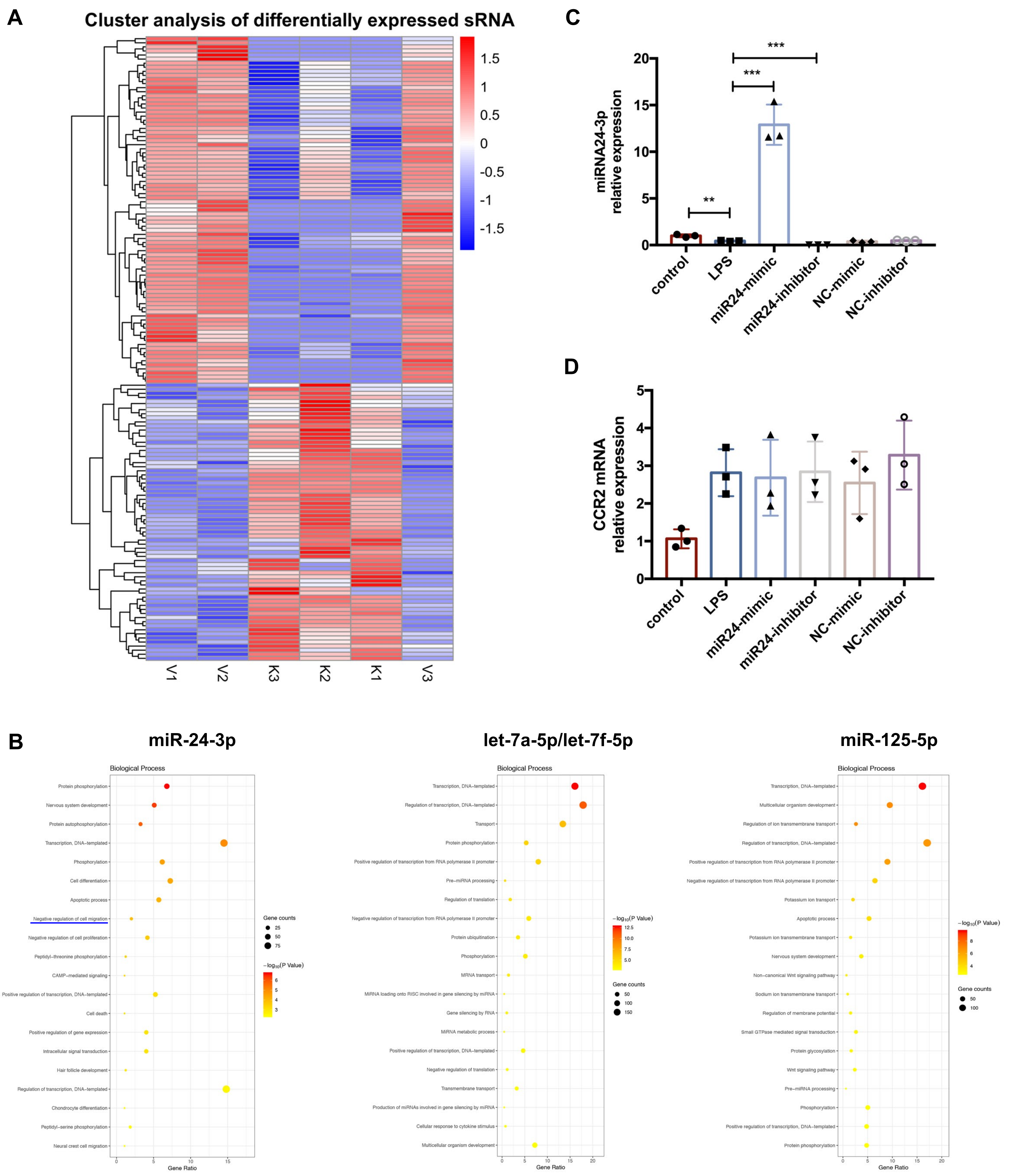

A
Cluster analysis of differentially expressed sRNA
1.5
1
0.5
0
-0.5
-1
-1.5
V1
V2
K3
K2
K1
V3
C
miRNA24-3p relative expression
20
15
10
5
0
**
***
***
control
LPS
miR24-mimic
miR24-inhibitor
NC-mimic
NC-inhibitor
D
CCR2 mRNA relative expression
5
4
3
2
1
0
control
LPS
miR24-mimic
miR24-inhibitor
NC-mimic
NC-inhibitor
B
miR-24-3p
Biological Process
Protein phosphorylation
Nervous system development
Protein autophosphorylation
Transcription, DNA−templated
Phosphorylation
Cell differentiation
Apoptotic process
Negative regulation of cell migration
Negative regulation of cell proliferation
Peptidyl−threonine phosphorylation
CAMP−mediated signaling
Positive regulation of transcription, DNA−templated
Cell death
Positive regulation of gene expression
Intracellular signal transduction
Hair follicle development
Regulation of transcription, DNA−templated
Chondrocyte differentiation
Peptidyl−serine phosphorylation
Neural crest cell migration
Gene Ratio
Gene counts
25
50
75
−log10(P Value)
let-7a-5p/let-7f-5p
Biological Process
Transcription, DNA−templated
Regulation of transcription, DNA−templated
Transport
Protein phosphorylation
Positive regulation of transcription from RNA polymerase II promoter
Pre−miRNA processing
Regulation of translation
Negative regulation of transcription from RNA polymerase II promoter
Protein ubiquitination
Phosphorylation
MRNA transport
MiRNA loading onto RISC involved in gene silencing by miRNA
Gene silencing by RNA
MiRNA metabolic process
Positive regulation of transcription, DNA−templated
Negative regulation of translation
Transmembrane transport
Production of miRNAs involved in gene silencing by miRNA
Cellular response to cytokine stimulus
Multicellular organism development
Gene Ratio
−log10(P Value)
12.5
10.0
7.5
5.0
Gene counts
50
100
150
miR-125-5p
Biological Process
Transcription, DNA−templated
Multicellular organism development
Regulation of ion transmembrane transport
Regulation of transcription, DNA−templated
Positive regulation of transcription from RNA polymerase II promoter
Negative regulation of transcription from RNA polymerase II promoter
Potassium ion transport
Apoptotic process
Potassium ion transmembrane transport
Nervous system development
Non−canonical Wnt signaling pathway
Sodium ion transmembrane transport
Regulation of membrane potential
Small GTPase mediated signal transduction
Protein glycosylation
Wnt signaling pathway
Pre−miRNA processing
Phosphorylation
Positive regulation of transcription, DNA−templated
Protein phosphorylation
Gene Ratio
−log10(P Value)
Gene counts
50
100

Figure S12

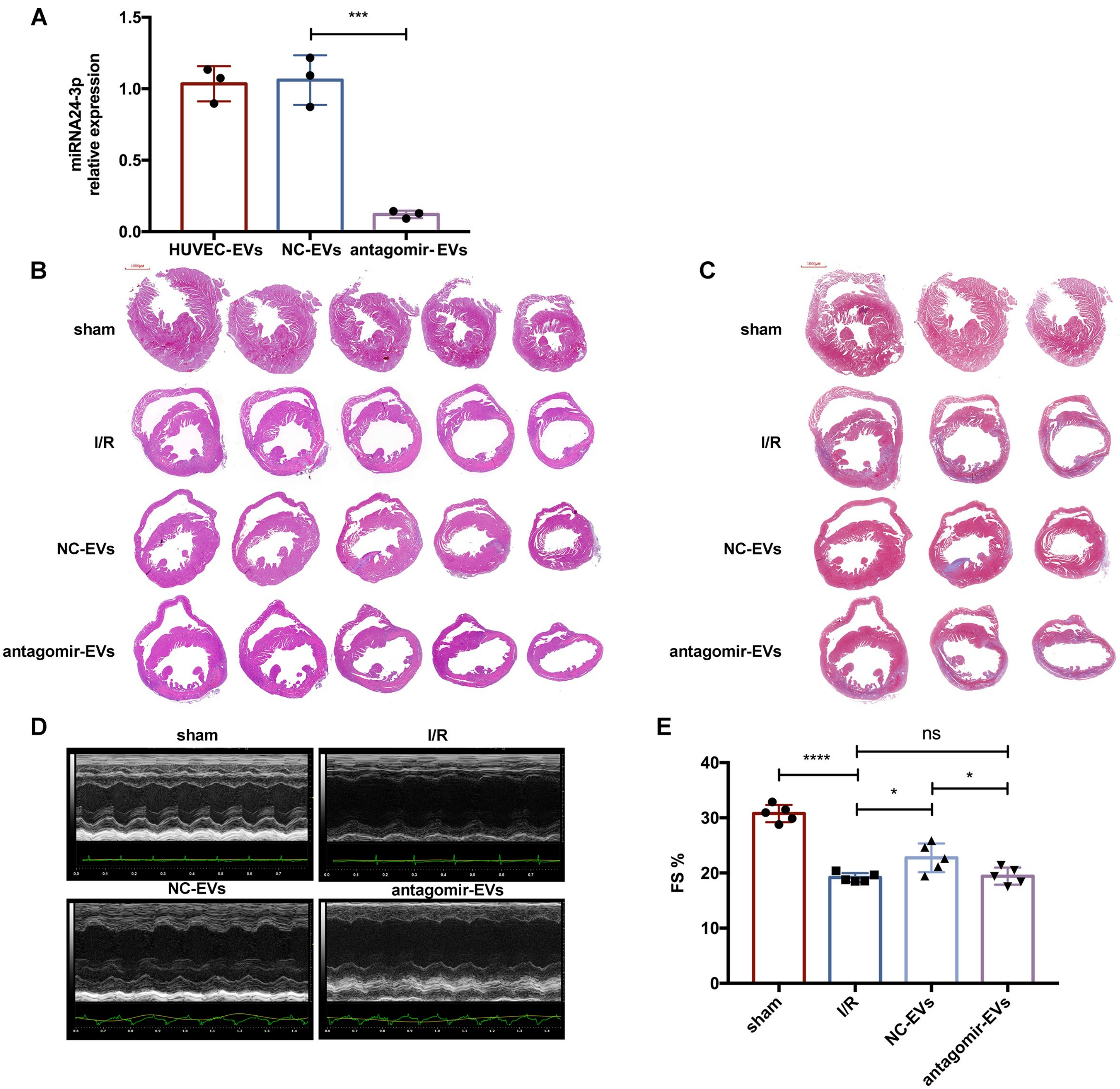
A
miRNA24-3p relative expression
1.5
1.0
0.5
0.0
***
HUVEC-EVs
NC-EVs
antagomir- EVs
B
sham
I/R
NC-EVs
antagomir-EVs
C
sham
I/R
NC-EVs
antagomir-EVs
D
sham
I/R
NC-EVs
antagomir-EVs
E
FS %
40
30
20
10
0
****
ns
*
*
sham
I/R
NC-EVs
antagomir-EVs

Figure S13

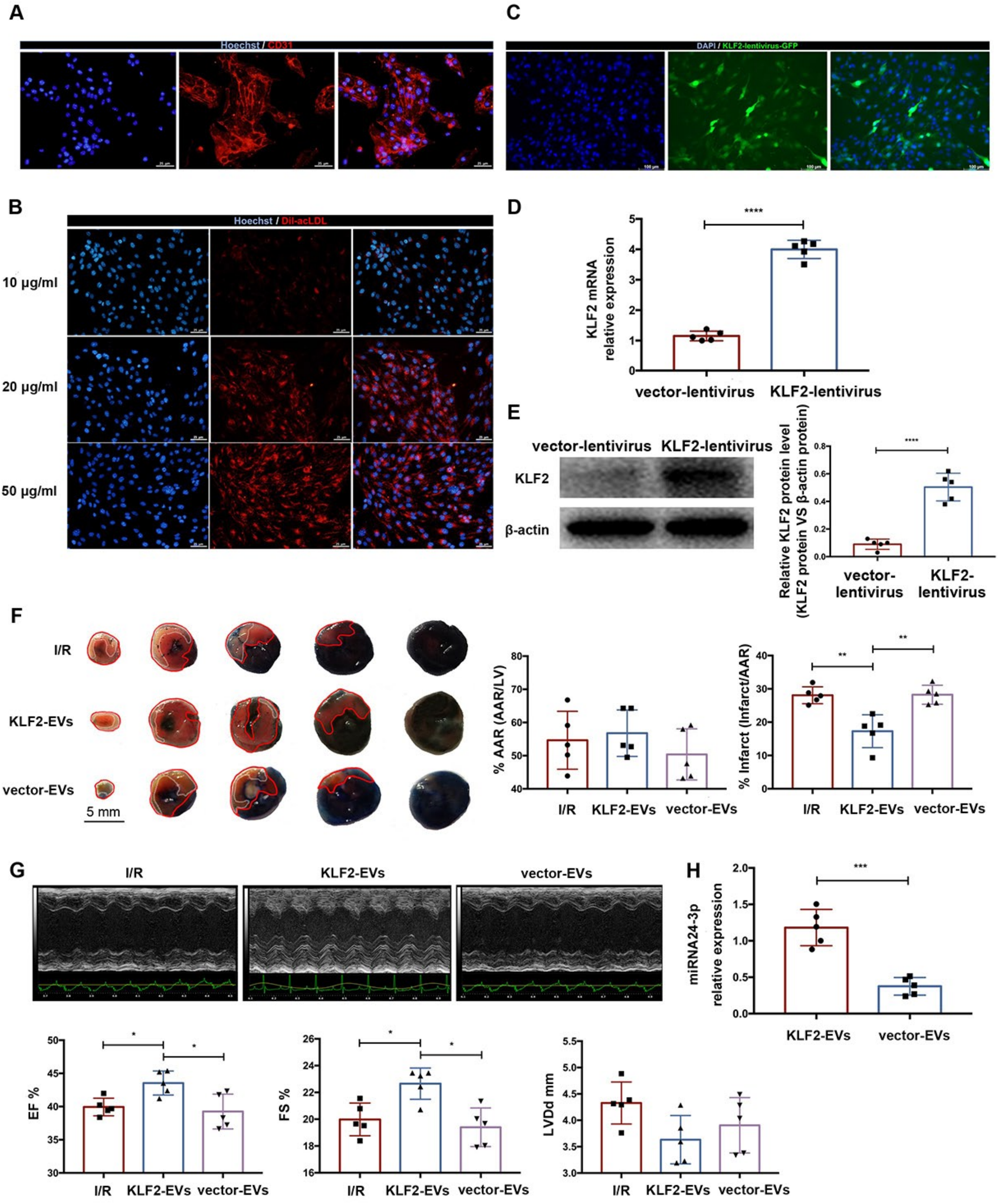
A
Hoechst / CD31
B
Hoechst / Dil-acLDL
10 μg/ml
20 μg/ml
50 μg/ml
C
DAPI / KLF2-lentivirus-GFP
D
KLF2 mRNA relative expression
vector-lentivirus
KLF2-lentivirus
****
E
vector-lentivirus
KLF2-lentivirus
KLF2
β-actin
Relative KLF2 protein level (KLF2 protein VS β-actin protein)
vector-lentivirus
KLF2-lentivirus
F
I/R
KLF2-EVs
vector-EVs
5 mm
% AAR (AAR/LV)
% Infarct (Infarct/AAR)
G
I/R
KLF2-EVs
vector-EVs
EF %
FS %
LVDd mm
H
miRNA24-3p relative expression
KLF2-EVs
vector-EVs

Supplementary Figure legend

Figure S1

The dose of extracellular vesicles (EVs) was determined according to our preliminary dose ranging experiment. Quantification of ejection fraction (EF) (A) and fractional shortening (FS) (B) in 3 days after myocardial I/R injury within sham-operated, PBS and KLF2-EVs at 1μg/g, 3μg/g or 10μg/g of body weight (n=5). Graphs depict mean ± SD. Statistical significance was measured via one-way ANOVA followed by Tukey's multiple comparisons test for multiple groups' comparison. *P < 0.05, ****P < 0.0001, ns= not significant.

Figure S2

HUVECs were activated into an anti-inflammatory phenotype by laminar shear stress with upregulation of KLF2. (A) Quantification of inflammatory cytokines with qRT-PCR, including IL-1β, IL-6, IL-10, TGF-β1 in control and laminar shear stress group (n=3). (B) Quantification of KLF2 mRNA with qRT-PCR in control and laminar shear stress group (n=3). (C) Quantification of WB to assess expression of KLF2 in control and laminar shear stress group (n=5). (D) Representative immunofluorescence staining of KLF2 (red) and HUVECs (DAPI blue) and quantification in control and laminar shear stress group (n=5). Scale bar=50 μm. Graphs depict mean ± SD. Statistical significance was measured via Student's t-test for two groups' comparison. *P < 0.05, **P < 0.01, ***P < 0.001.

Figure S3

Transfecting HUVECs with a lentivirus vector encoding KLF2 (Lv-KLF2) remarkably increased KLF2 expression. (A) Representative fluorescence staining of KLF2 (GFP green) in KLF2-transduced HUVECs compared with empty vector-transduced HUVECs. Scale bar=250 μm. (B) Quantification of KLF2 mRNA with qRT-PCR in KLF2-transduced HUVECs compared with empty vector-transduced HUVECs (n=3). Graphs depict mean ± SD. Statistical significance was measured via Student's t-test for two groups' comparison. **P < 0.01.

Figure S4

Extracellular vesicles (EVs) from KLF2-transduced HUVECs ameliorated myocardial ischemia/reperfusion (I/R) injury and alleviated inflammation. (A) Representative echocardiography M-mode images and quantification of fractional shortening (FS) within sham-operated, PBS or KLF2-EVs treated mice in 3 days (A, B) and 2 weeks (C, D) following

myocardial I/R injury (n=5). (E) Representative images of H.E. staining to assess infarct area in different treatment groups. Scale bar=1000 μm. (F) Representative images of Masson trichrome staining to assess scar area in different treatment group. Scale bar=1000 μm. (G) Quantification of mRNA of IL-1β, IL-10, iNOS, Arg-1 using qRT-PCR in different treatment groups (n=5). Graphs depict mean ± SD. Statistical significance was measured via one-way ANOVA followed by Tukey's multiple comparisons test for multiple groups' comparison. *$P < 0.05$, **$P < 0.01$, ***$P < 0.001$, ****$P < 0.0001$.

Figure S5

Extracellular vesicles (EVs) derived from empty vector-transduced HUVECs could not ameliorate myocardial I/R injury and alleviated inflammation level. (A) Representative images of hearts with Evans blue/TTC staining from mice 3 days following treatment with PBS or vector-EVs. Area-at-risk (AAR): red line; infarct size (IS): white dotted line. Scale bar=5mm. Quantitative analysis of the percentage AAR (B) and percentage infarct of hearts (C) in (A) (n=5). (D) Representative echocardiography M-mode images and quantification of ejection fraction (EF) (E) and fractional shortening (FS) (F) within sham-operated, PBS or vector-EVs treated mice in 3 days after myocardial I/R injury (n=5). Concentration of cytokines IL-1β (G) and IL-10 (H) detected by ELISA in serum of sham-operated, PBS or vector-EVs treated mice (n=5). Graphs depict mean ± SD. Statistical significance was measured via one-way ANOVA followed by Tukey's multiple comparisons test for multiple groups' comparison. **$P < 0.01$, ****$P < 0.0001$, ns= not significant.

Figure S6

Systemic depletion of monocytes/macrophages obliterated the efficacy of KLF2-EVs therapy. (A) Representative flow cytometry plots and quantification of Mo/Mø (CD11b+), $Ly6C_{high}$ Mo/Mø ($CD11b+Ly6C_{high}$) and $Ly6C_{low}$ Mo/Mø ($CD11b+Ly6C_{low}$) in the heart, peripheral blood and spleen from Cl_2MDP and PBS treated mice (n=3). Representative echocardiography M-mode images (B) and quantification of fractional shortening (FS) (C) in sham-operated, PBS or KLF2-EVs treated mice 3 days following myocardial I/R injury and Cl_2MDP treatment (n=5). Graphs depict mean ± SD. Statistical significance was measured via Student's t-test for two groups' comparison, one-way ANOVA followed by Tukey's multiple comparisons test for multiple groups' comparison and two-way ANOVA followed by Bonferroni's multiple comparisons test

for comparison between different groups in different cell subtypes. *P < 0.05, ***P < 0.001, ****P < 0.0001, ns= not significant.

Figure S7

KLF2-EVs did not affect the apoptosis and polarization of Ly6C$_{high}$ Mo/Mø in vitro. (A) Representative flow cytometry plots showing apoptosis and quantification of Annexin V+PI+ cells proportion in RAW264.7 cells with treatment of PBS or KLF2-EVs in 6 hours hypoxia followed by reoxygenation treatment (n=3). (B) Representative flow cytometry plots showing polarization and quantification of iNOS+CD206- or CD206+iNOS- cells proportion in RAW264.7 cells with treatment of PBS or KLF2-EVs in 6 hours lipopolysaccharide (100ng/ml) stimulation (n=3). Graphs depict mean ± SD. Statistical significance was measured via Student's t-test for two groups' comparison, and two-way ANOVA followed by Bonferroni's multiple comparisons test for comparison between different groups in different cell subtypes. ****P < 0.0001.

Figure S8

KLF2-EVs did not inhibited Ly6C$_{high}$ monocytes recruitment from spleen. Representative echocardiography M-mode images (A) and quantification of fractional shortening (FS) (B) in sham-operated, PBS or KLF2-EVs treated mice 3 days after myocardial I/R injury and splenectomy (n=5). Quantification of ejection fraction (EF) (C) and fractional shortening (FS) (D) in 3 days after myocardial I/R injury in sham, splenectomy, I/R, I/R+splenectomy, I/R+KLF2-EVs and I/R+splenectomy+KLF2-EVs (n=5). Graphs depict mean ± SD. Statistical significance was measured via one-way ANOVA followed by Tukey's multiple comparisons test for multiple groups' comparison. *P < 0.05, **P < 0.01, ****P < 0.0001, ns= not significant.

Figure S9

KLF2-EVs inhibited Ly6C$_{high}$ monocytes recruitment from bone marrow in vivo. (A) Quantification of mRNA of Agtr1a within spleen tissue in sham-operated, PBS or KLF2-EVs treated mice following myocardial I/R injury and splenectomy (n=5). (B) Representative images of WB and quantification to assess expression of Agtr1a within spleen tissues in sham-operated, PBS or KLF2-EVs treated mice following myocardial I/R injury and splenectomy (n=5). (C) Quantification of mRNA of CCR2 and CX3CR1 using qRT-PCR in sham-operated, PBS or KLF2-EVs treated mice following myocardial I/R injury (n=5). (D) Representative immunohistochemical staining and quantification of CX3CR1 in hearts heart in sham-operated,

PBS or KLF2-EVs treated mice following myocardial I/R injury (n=5). Scale bar=50 μm. (E) Concentration of cytokines CCL2 and CX3CL1 detected by ELISA in sham-operated, PBS or KLF2-EVs treated mice serum following myocardial I/R injury (n=5). Graphs depict mean ± SD. Statistical significance was measured via one-way ANOVA followed by Tukey's multiple comparisons test for multiple groups' comparison and two-way ANOVA followed by Bonferroni's multiple comparisons test for comparison between different groups in different subgroups. *P < 0.05, **P < 0.01, ***P < 0.001, ****P < 0.0001.

Figure S10

KLF2-EVs restrained RAW264.7 cells migration in vitro. (A) Representative images of RAW264.7 cells in Transwell experiment with treatment of PBS, MCP-1, KLF2-EVs and vector-EVs, and quantification of number of migrated cells (n=5). Scale bar=50 μm. (B) Representative images of RAW264.7 cells in scratch wound healing assay with treatment of PBS, MCP-1, KLF2-EVs and vector-EVs, and quantification of length percentage of cells migration (n=5). Scale bar=100 μm. (C) Quantification of mRNA of CCR2 of RAW264.7 cells using qRT-PCR in control, LPS, KLF2-EVs and vector-EVs groups (n=3). (D) Representative images of WB and quantification to assess expression of CCR2 of RAW264.7 cells in control, LPS, KLF2-EVs and vector-EVs groups (n=3). (E) Quantification of mRNA of CX3CR1 of RAW264.7 cells using qRT-PCR in control, LPS, KLF2-EVs and vector-EVs groups (n=3). (F) Representative images of WB and quantification to assess expression of CX3CR1 of RAW264.7 cells in control, LPS, KLF2-EVs and vector-EVs groups (n=3). Graphs depict mean ± SD. Statistical significance was measured via one-way ANOVA followed by Tukey's multiple comparisons test for multiple groups' comparison. *P < 0.05, **P < 0.01, ***P < 0.001, ****P < 0.0001, ns= not significant.

Figure S11

miR-24-3p regulated KLF2-EV-mediated Ly6C$_{high}$ monocyte recruitment. (A) Heatmap plot of differentially expressed miRNA in KLF2-EVs and vector-EVs cluster. (B) Bubble map of pathway enrichment achieved from miRNA target genes with GO enrichment analysis for 4 miRNAs. (C) Quantification of miRNA21-3p using qRT-PCR in RAW264.7 cells treated of PBS, LPS, miR-24-3p mimic/inhibitor and NC mimic/inhibitor (n=3). (D) Quantification of CCR2 mRNA using qRT-PCR in RAW264.7 cells treated of PBS, LPS, miR-24-3p mimic/inhibitor and NC mimic/inhibitor (n=3). Graphs depict mean ± SD. Statistical significance was measured via

Student's t-test for two groups' comparison and one-way ANOVA followed by Tukey's multiple comparisons test for multiple groups' comparison. $^{**}P < 0.01$, $^{***}P < 0.001$.

Figure S12

miR-24-3p antagomir abrogated the effect of KLF2-EVs on ameliorating myocardial I/R injury. (A) Quantification of miR-24-3p in HUVEC-EVs, NC-EVs and antagomir-EVs (n=3). (B) Representative images of H.E. staining to assess infarct area in sham-operated, I/R+PBS, I/R+NC-EVs or I/R+antagomir-EVs treated mice 3 days after myocardial I/R injury. Scale bar=1000 μm. (C) Representative images of Masson trichrome staining to assess scar area in sham-operated, I/R+PBS, I/R+NC-EVs or I/R+antagomir-EVs treated mice 3 days following myocardial I/R injury. Scale bar=1000 μm. Representative echocardiography M-mode images (D) and quantification of fractional shortening (FS) (E) in sham-operated, I/R+PBS, I/R+NC-EVs or I/R+antagomir-EVs treated mice 3 days after myocardial I/R injury (n=5). Graphs depict mean ± SD. Statistical significance was measured via one-way ANOVA followed by Tukey's multiple comparisons test for multiple groups' comparison. $^{**}P < 0.01$, $^{***}P < 0.001$, $^{****}P < 0.0001$, ns= not significant.

Figure S13

KLF2-MCAECs-derived EVs ameliorated myocardial I/R injury and were rich in miR-24-3p. (A) Fluorescence images show that CD31 staining in red color in MCAECs, and the nucleus was stained by Hoechst in blue color. Scale bar=25μm. (B) Fluorescence images show that acLDL uptake of red color in MCAECs, and the nucleus was stained by Hoechst in blue color. Scale bar=25μm. (C) Representative fluorescence staining of KLF2 (GFP green) in KLF2-transduced MCAECs compared with empty vector-transduced MCAECs. Scale bar=100μm. (D) Quantification of KLF2 mRNA with qRT-PCR in KLF2-transduced MCAECs compared with empty vector-transduced MCAECs (n=5). (E) Representative images and quantification of WB to assess expression of KLF2 in KLF2-transduced MCAECs among with empty vector-transduced MCAECs (n=5). (F) Representative images of hearts with Evans blue/TTC staining from mice 3 days following treatment with PBS, KLF2-EVs or vector-EVs. Area-at-risk (AAR): red line; infarct size (IS): white dotted line. Scale bar=5mm. Quantitative analysis of the percentage of AAR/left ventricle and percentage of infarct/AAR (n=5). (G) Representative echocardiography M-mode images and quantification of ejection fraction (EF), fractional shortening (FS) and left

ventricular end-diastolic diameter (LVDd) within PBS, KLF2-EVs or vector-EVs treated mice in 3 days following myocardial I/R injury (n=5). (H) Quantification of miR-24-3p in KLF2-EVs compared to vector-EVs derived from MCAECs (n=5). Graphs depict mean ± SD. Statistical significance was measured via Student's t-test for two groups' comparison and one-way ANOVA followed by Tukey's multiple comparisons test for multiple groups' comparison. $*P < 0.05$, $**P < 0.01$, $***P < 0.001$, $****P < 0.0001$.

Table S1. Nanoparticle tracking analysis of extracellular vesicles (EVs)

	laminar-EVs	WT-EVs	KLF2-EVs	vector-EVs
Sample Name	A186-01-01	A186-01-02	A186-02	A186-03
Volume (ul)	200	200	200	200
Median Diameter (nm)	97.1	95.1	90.8	98.9
Diameter Peak (nm)	81.4	80.3	78.6	86.2
Percentage (%)	97.4	97.5	99	98.5
Concentration (Particles / mL)	7.0*10^9	2.7*10^10	3.8*10^10	5.2*10^10

Table S2. Echocadiography of I/R mice-1						
	3d			14d		
	sham	I/R	KLF2-EVs	sham	I/R	KLF2-EVs
IVS;d	0.71±0.01	0.78±0.04	0.77±0.03	0.83±0.08	0.72±0.06	0.72±0.04
IVS;s	1.16±0.03	1.04±0.08	1.11±0.05	1.31±0.17	1.02±0.07	1.07±0.07
LVID;d	3.57±0.06	3.33±0.13	3.65±0.21	3.24±0.19	3.96±0.18	3.54±0.12
LVID;s	2.35±0.08	2.59±0.10	2.78±0.14	2.15±0.15	2.69±0.21	2.46±0.09
LVPW;d	0.71±0.01	0.76±0.03	0.85±0.09	0.79±0.05	0.72±0.07	0.75±0.03
LVPW;s	1.21±0.08	1.05±0.05	1.05±0.06	1.34±0.16	1.12±0.12	1.15±0.07
EF	64.63±1.35	44.15±2.60	49.98±0.78	67.60±2.42	53.06±1.16	56.79±0.96
FS	34.73±1.21	21.35±1.55	25.10±1.16	34.88±2.29	26.74±0.87	29.78±1.08
LV Mass	80.44±2.15	832.74±6.41	99.82±7.80	83.94±6.60	90.13±10.11	83.31±9.53
LV Mass (Corrected)	63.98±2.79	67.77±5.18	80.37±5.90	68.23±5.03	72.17±8.25	66.29±7.60
LV Vol;d	53.14±1.90	46.86±4.01	56.46±8.12	45.26±4.70	60.67±11.21	50.24±4.96
LV Vol;s	17.62±2.10	26.13±1.25	27.91±3.50	15.09±2.12	23.65±3.79	21.00±2.36

IVS;d: end-diastolic interventricular septal thickness; IVS;s: end-systolic interventricular septal thickness; LVID;d: left ventricular end-diastolic diameter; LVID;s: left ventricular end-systolic diameter; LVPW;d: end-diastolic left ventricular posterior wall thickness; LVPW;s: end-systolic left ventricular posterior wall thickness; EF: left ventricular ejection fraction; FS: left ventricular fractional shortening; LV Vol;d: end-diastolic left ventricular volume; LV Vol;s =end-systolic left ventricular volume.

Table S3. Echocadiography of I/R mice-2			
	sham	I/R	vector-EVs
IVS;d	0.71±0.01	0.78±0.04	0.74±0.05
IVS;s	1.16±0.03	1.04±0.08	1.07±0.04
LVID;d	3.57±0.06	3.33±0.13	3.65±0.21
LVID;s	2.35±0.08	2.59±0.10	2.91±0.23
LVPW;d	0.71±0.01	0.76±0.03	0.80±0.04
LVPW;s	1.21±0.08	1.05±0.05	1.03±0.09
EF	64.63±1.35	44.15±2.60	46.89±0.88
FS	34.73±1.21	21.35±1.55	22.84±0.87
LV Mass	80.44±2.15	832.74±6.41	98.74±8.98
LV Mass (Corrected)	63.98±2.79	67.77±5.18	78.06±6.67
LV Vol;d	53.14±1.90	46.86±4.01	56.58±8.19
LV Vol;s	17.62±2.10	26.13±1.25	30.00±3.85

IVS;d: end-diastolic interventricular septal thickness; IVS;s: end-systolic interventricular septal thickness; LVID;d: left ventricular end-diastolic diameter; LVID;s: left ventricular end-systolic diameter; LVPW;d: end-diastolic left ventricular posterior wall thickness; LVPW;s: end-systolic left ventricular posterior wall thickness; EF: left ventricular ejection fraction; FS: left ventricular fractional shortening; LV Vol;d: end-diastolic left ventricular volume; LV Vol;s =end-systolic left ventricular volume.

Table S4. Echocadiography of I/R+Cl_2MDP mice			
	sham	I/R	KLF2-EVs
IVS;d	0.74±0.08	0.81±0.04	0.73±0.05
IVS;s	1.10±0.07	1.25±0.10	1.13±0.16
LVID;d	3.79±0.17	3.90±0.22	3.75±0.33
LVID;s	2.50±0.17	3.05±0.23	2.81±0.22
LVPW;d	0.74±0.06	1.12±0.23	0.68±0.09
LVPW;s	1.25±0.12	1.22±0.12	0.87±0.16
EF	63.95±2.17	44.87±1.55	45.95±3.80
FS	34.75±2.20	23.13±1.16	21.98±3.66
LV Mass	93.91±5.96	124.90±15.68	80.06±4.93
LV Mass (Corrected)	76.86±5.37	96.39±14.08	64.72±1.55
LV Vol;d	64.19±4.62	66.68±5.64	52.81±5.39
LV Vol;s	24.68±3.42	38.78±1.58	30.03±5.83

IVS;d: end-diastolic interventricular septal thickness; IVS;s: end-systolic interventricular septal thickness; LVID;d: left ventricular end-diastolic diameter; LVID;s: left ventricular end-systolic diameter; LVPW;d: end-diastolic left ventricular posterior wall thickness; LVPW;s: end-systolic left ventricular posterior wall thickness; EF: left ventricular ejection fraction; FS: left ventricular fractional shortening; LV Vol;d: end-diastolic left ventricular volume; LV Vol;s =end-systolic left ventricular volume.

Table S5. Echocadiography of I/R+splenectomy mice			
	sham	I/R	KLF2-EVs
IVS;d	0.67±0.03	0.63±0.07	0.72±0.05
IVS;s	1.13±0.10	0.91±0.06	1.06±0.07
LVID;d	3.86±0.05	4.14±0.24	4.03±0.28
LVID;s	2.61±0.06	3.27±0.16	2.97±0.32
LVPW;d	0.67±0.05	0.67±0.03	0.78±0.04
LVPW;s	1.07±0.08	0.98±0.01	1.15±0.15
EF	62.51±1.80	46.06±2.03	51.79±3.40
FS	33.35±1.63	23.32±1.07	27.41±2.69
LV Mass	87.94±3.67	92.40±4.42	102.88±14.71
LV Mass (Corrected)	70.53±2.56	73.54±3.80	83.08±11.47
LV Vol;d	64.23±2.35	79.05±7.12	74.09±11.55
LV Vol;s	24.56±0.59	41.95±3.97	36.58±8.02

IVS;d: end-diastolic interventricular septal thickness; IVS;s: end-systolic interventricular septal thickness; LVID;d: left ventricular end-diastolic diameter; LVID;s: left ventricular end-systolic diameter; LVPW;d: end-diastolic left ventricular posterior wall thickness; LVPW;s: end-systolic left ventricular posterior wall thickness; EF: left ventricular ejection fraction; FS: left ventricular fractional shortening; LV Vol;d: end-diastolic left ventricular volume; LV Vol;s =end-systolic left ventricular volume.

Table S6. miRNA array analysis in KLF2-EVs and vector-EVs

sRNA	KLF2_EVs_readcount	vec_EVs_readcount	log2FoldChange	pval	padj
hsa-miR-518b	80.95709263	0	9.1348	0.00031263	0.0033987
hsa-miR-519d-3p	52.88340286	0	8.5204	0.0010403	0.009425
hsa-miR-6727-5p	72.67122968	1.296713245	5.9137	0.00012425	0.0014774
hsa-miR-150-5p	153.1353324	5.681829032	4.7631	0.0026838	0.019829
hsa-miR-92a-1-5p	967.3590434	59.0541502	4.0335	5.68E-23	8.65E-21
hsa-miR-223-3p	110.2942226	9.124587196	3.6096	0.00090707	0.0084181
hsa-miR-365b-5p	4160.032334	359.8232112	3.5324	1.87E-19	1.42E-17
hsa-miR-365a-5p	5849.715315	506.6019167	3.5295	4.10E-09	1.11E-07
hsa-miR-184	557.4517819	69.2383072	3.0087	3.75E-05	0.000529
hsa-miR-340-3p	240.1936392	33.84088965	2.8324	1.06E-06	2.08E-05
hsa-miR-125a-5p	5556.897318	784.7020719	2.8244	4.33E-19	2.99E-17
hsa-miR-30c-1-3p	202.7304376	31.48959463	2.6889	3.56E-08	8.20E-07
hsa-miR-30c-2-3p	776.351279	133.635185	2.5388	9.27E-16	5.43E-14
hsa-miR-671-5p	146.2630296	25.28622527	2.5268	0.0006096	0.006104
hsa-miR-130b-5p	1223.022147	212.5728201	2.5242	4.74E-07	9.75E-06
hsa-miR-24-3p	10578.72169	1921.136663	2.4611	7.47E-06	0.0001184
hsa-let-7a-5p	106337.7701	19852.97182	2.4212	3.94E-29	3.00E-26
hsa-miR-139-3p	1609.632639	310.2795723	2.3765	2.11E-20	2.25E-18
hsa-miR-9-5p	1154.798936	229.3359774	2.3316	1.48E-09	4.49E-08
hsa-let-7f-5p	45027.84826	9777.767102	2.2032	1.18E-25	3.00E-23
hsa-miR-125b-5p	11095.05492	2473.303988	2.1655	6.86E-12	2.90E-10
hsa-let-7d-3p	5594.509328	1253.568918	2.158	5.29E-17	3.36E-15
hsa-miR-1-3p	2239.233171	537.780417	2.057	1.12E-13	5.32E-12
hsa-miR-451a	3199.408495	805.136868	1.9907	0.00011182	0.001395
hsa-miR-10a-5p	115230.0574	29243.22848	1.9783	5.16E-21	6.54E-19
hsa-miR-1180-3p	47578.44509	12122.6495	1.9726	4.74E-24	9.02E-22
hsa-miR-139-5p	2338.883667	618.567675	1.9195	7.27E-14	3.80E-12
hsa-miR-411-3p	131.3441134	37.30382501	1.8106	0.0031701	0.021479
hsa-miR-486-3p	7165.011169	2073.508261	1.7891	2.37E-20	2.25E-18
hsa-miR-486-5p	7195.656484	2084.338877	1.7878	6.38E-20	5.40E-18
hsa-miR-145-3p	281.5260036	83.10638487	1.7613	0.0019271	0.014965
hsa-let-7e-5p	11570.90572	3500.307626	1.7249	1.33E-10	4.59E-09
hsa-miR-206	2885.252003	901.4361224	1.6784	8.29E-05	0.0010875
hsa-miR-505-3p	605.5271149	194.8521392	1.6376	6.41E-06	0.00010603
hsa-miR-208b-3p	399.0121092	132.3047424	1.59	0.0017136	0.014022
hsa-miR-92b-5p	401.5562671	140.8118773	1.5113	0.0018873	0.014815
hsa-miR-3184-5p	23324.66229	8616.595143	1.4367	5.84E-09	1.48E-07
hsa-miR-423-3p	23309.93772	8612.632029	1.4365	5.71E-09	1.48E-07
hsa-miR-125b-1-3p	827.1158035	317.6923949	1.3811	0.0002855	0.0031951
hsa-miR-1293	638.2774422	252.3701947	1.3387	0.0070939	0.040754
hsa-miR-10b-5p	314971.7206	125113.9325	1.332	1.50E-11	6.00E-10
hsa-miR-26b-5p	674.4738077	267.8411458	1.332	0.0012512	0.01082
hsa-miR-499a-5p	971.1535769	385.6909175	1.3314	3.67E-05	0.00052764
hsa-miR-499b-3p	971.1535769	385.6909175	1.3314	3.67E-05	0.00052764
hsa-miR-122-5p	872572.3838	353458.405	1.3037	0.007675	0.041719
hsa-miR-122b-3p	872572.3838	353458.405	1.3037	0.007675	0.041719
hsa-miR-98-5p	1030.474219	421.286741	1.2894	3.89E-06	6.87E-05
hsa-miR-487a-5p	804.9748311	335.414273	1.2641	1.07E-05	0.00016628
hsa-miR-615-3p	258.1907638	107.8436425	1.2597	0.0031286	0.021479
hsa-miR-100-5p	227907.3028	101455.4208	1.1676	2.08E-13	9.32E-12
hsa-miR-412-5p	9154.764001	4138.574509	1.1453	2.32E-09	6.80E-08
hsa-let-7d-5p	1232.55479	560.6098291	1.1372	0.0018884	0.014815
hsa-miR-342-5p	1229.572916	562.8930135	1.1268	0.0073665	0.04122
hsa-let-7g-5p	5860.982508	2716.638332	1.1093	6.19E-06	0.00010464
hsa-miR-142-5p	834.065478	392.3905428	1.0879	0.0010937	0.0097917
hsa-miR-425-5p	876.1026116	416.2585547	1.0741	0.0098192	0.048884

hsa-let-7i-5p	163986.7463	84241.54855	0.96098	3.50E-07	7.40E-06
hsa-miR-195-3p	1377.610095	720.2856309	0.93519	0.0025303	0.019065
hsa-miR-126-3p	90064.93347	49378.95011	0.86706	6.89E-07	1.38E-05
hsa-miR-143-3p	14179.19695	7797.054007	0.86266	1.45E-05	0.00021591
hsa-miR-222-3p	803420.4619	492844.4203	0.70502	0.00039287	0.0040955
hsa-miR-6529-5p	2869.178581	1806.079474	0.66777	0.008362	0.0445
hsa-miR-127-3p	12443.99946	7910.101469	0.65372	0.0076476	0.041719
hsa-miR-155-5p	6360.993633	4308.973385	0.56199	0.0056673	0.034503
hsa-miR-224-5p	18028.03968	12620.91053	0.51451	0.0096936	0.048884
hsa-miR-3184-3p	604831.5734	428001.5495	0.49892	0.0029892	0.021071
hsa-miR-423-5p	604831.5734	428004.4671	0.49891	0.0029904	0.021071
hsa-miR-128-3p	37909.46805	28183.98833	0.42767	0.0057174	0.034531

Table S7. Echocadiography of I/R+NC/miR24-3p-antgomir-EVs mice				
	sham	I/R	NC-EVs	antagomir-EVs
IVS;d	0.70±0.03	0.74±0.08	0.53±0.13	0.75±0.03
IVS;s	1.12±0.06	1.00±0.09	0.78±0.08	1.12±0.16
LVID;d	3.99±0.07	3.92±0.40	3.78±0.31	4.05±0.42
LVID;s	2.76±0.07	3.21±0.37	3.28±0.22	3.44±0.09
LVPW;d	0.71±0.05	0.71±0.02	0.74±0.06	0.85±0.22
LVPW;s	1.05±0.07	1.01±0.06	0.958±0.0	0.98±0.18
EF	60.06±1.89	39.53±1.54	45.98±2.21	39.61±3.76
FS	30.79±1.56	19.18±0.82	22.75±2.592	19.43±1.56
LV Mass	98.17±8.70	91.40±7.16	95.12±4.75	105.04±12.26
LV Mass (Corrected)	78.02±5.42	71.85±8.72	79.77±5.07	81.58±8.22
LV Vol;d	67.90±1.83	63.23±12.44	76.66±7.18	73.45±1.18
LV Vol;s	27.70±1.03	38.51±8.10	42.35±3.33	47.25±8.37

IVS;d: end-diastolic interventricular septal thickness; IVS;s: end-systolic interventricular septal thickness; LVID;d: left ventricular end-diastolic diameter; LVID;s: left ventricular end-systolic diameter; LVPW;d: end-diastolic left ventricular posterior wall thickness; LVPW;s: end-systolic left ventricular posterior wall thickness; EF: left ventricular ejection fraction; FS: left ventricular fractional shortening; LV Vol;d: end-diastolic left ventricular volume; LV Vol;s =end-systolic left ventricular volume.

Table S8. Echocadiography of I/R+mouse-KLF2/vector-EVs mice

	I/R	KLF2-EVs	vector-EVs
IVS;d	0.84±0.10	0.75±0.12	0.81±0.06
IVS;s	1.21±0.11	0.98±0.12	1.02±0.13
LVID;d	4.33±0.40	3.63±0.46	3.91±0.52
LVID;s	3.52±0.30	3.38±0.17	3.49±0.12
LVPW;d	0.81±0.09	0.79±0.02	0.86±0.23
LVPW;s	1.15±0.07	0.99±0.02	1.02±0.21
EF	39.94±1.33	43.55±1.81	39.25±2.63
FS	19.98±1.22	22.66±1.17	19.39±1.44
LV Mass	99.30±8.26	99.42±4.57	102.21±8.98
LV Mass (Corrected)	79.58±8.27	89.19±4.98	92.31±7.82
LV Vol;d	65.32±10.21	75.11±6.81	71.54±1.8
LV Vol;s	41.15±8.04	42.53±3.83	45.52±7.98

IVS;d: end-diastolic interventricular septal thickness; IVS;s: end-systolic interventricular septal thickness; LVID;d: left ventricular end-diastolic diameter; LVID;s: left ventricular end-systolic diameter; LVPW;d: end-diastolic left ventricular posterior wall thickness; LVPW;s: end-systolic left ventricular posterior wall thickness; EF: left ventricular ejection fraction; FS: left ventricular fractional shortening; LV Vol;d: end-diastolic left ventricular volume; LV Vol;s =end-systolic left ventricular volume

Table S9. Primer sequences for quantitative reverse transcriptase polymerase chain reaction (qRT-PCR)

gene	species	primer sequences	
		forward (5' -> 3')	reverse (5' -> 3')
CX3CR1	mouse	GAGTATGACGATTCTGCTGAGG	CAGACCGAACGTGAAGACGAG
CCR2	mouse	GGAGAAAAGCCAACTCCTTC	AGGCAGTTGCAAAGGTACTG
GAPDH	mouse	AGAACATCATCCCTGCCTCTACT	AGAACATCATCCCTGCCTCTACT
Agtr1α	mouse	AACAGCTTGGTGGTGATCGTC	CATAGCGGTATAGACAGCCCA
TNFα	mouse	AACTCCAGGCGGTGCCTATG	TCCAGCTGCTCCTCCACTTG
IL-10	mouse	ACTCTTCACCTGCTCCACTG	GCTATGCTGCCTGCTCTTAC
iNOS	mouse	TCACCTTCGAGGGCAGCCGA	TCCGTGGCAAAGCGAGCCAG
Arg1	mouse	CCAGATGTACCAGGATTCTC	AGCAGGTAGCTGAAGGTCTC
IL-1β	mouse	AGCTTCAGGCAGGCAGTATC	TCATCTCGGAGCCTGTAGTG
IL-6	mouse	AAGTCCGGAGAGGAGACTTC	TGGATGGTCTTGGTCCTTAG
CD206	mouse	CTGCAGATGGGTGGGTTATT	GGCATTGATGCTGCTGTTATG
TGFβ1	mouse	CGGAGAGCCCTGGATACCA	GCCGCACACAGCAGTTCTT
ICAM-1	human	TTGGGCATAGAGACCCCGTT	GCACATTGCTCAGTTCATACACC
KLF2	human	GCACGCACACAGGTGAGAAG	ACCAGTCACAGTTTGGGAGGG
TGF-β1	human	GGCCAGATCCTGTCCAAGC	GTGGGTTTCCACCATTAGCAC
IL-10	human	GACTTTAAGGGTTACCTGGGTTG	TCACATGCGCCTTGATGTCTG
TNFα	human	CCTCTCTCTAATCAGCCCTCTG	GAGGACCTGGGAGTAGATGAG
IL-1β	human	ATGATGGCTTATTACAGTGGCAA	GTCGGAGATTCGTAGCTGGA
IL-6	human	ACTCACCTCTTCAGAACGAATTG	CCATCTTTGGAAGGTTCAGGTTG
GAPDH	human	GGAGCGAGATCCCTCCAAAAT	GGCTGTTGTCATACTTCTCATGG
miR24-3p	mouse/human	AGTGGCTCAGTTCAGCA	GTCCAGTTTTTTTTTTTTTTTCTGTTC
U6	mouse/human	CGCTTCGGCAGCACATATAC	AAATATGGAACGCTTCACGA

Table S10. Primary antibodies used for western blotting			
Antibody	Source	Catalog number	Host species
CD9	Abcam	ab92726	rabbit
ALIX	Abcam	ab186429	rabbit
TSG101	Abcam	ab125011	rabbit
CD63	SBI	EXOAB-CD63A-1	rabbit
GAPDH	MultiSciences	ab011	mouse
CX3CR1	Proteintech	13885-1-AP	rabbit
CCR2	Abcam	ab203128	mouse
Agtr1α	Proteintech	25343-1-AP	rabbit
KLF2	Abcam	ab139699	rabbit
β-actin	Servicebio	GB11001	rabbit

Certificate of Analysis (COA)

Product: Exosome Depleted Fetal Bovine Serum
Catalog No.: C38010050, C38010100
Lot No.: 0054819
Size: 50ml, 100ml
Storage Condition: -10~-20℃
Date of manufacture: 2019.11.29
Expiration Date: 2024.02.28
Date of report: 2020.01.07

Reference	Test	Specification	Result	Pass
1	pH	7.0-8.0	7.75	Ok
2.	Osmolality(mOsm/kg)	270-345	306	Ok
3.	Sterility testing	Negative	Negative	Ok
4	Mycoplasma testing	Negative	Negative	Ok
5	Endotoxin testing(EU/ml)	≤10	<0.5	Ok
6	Plating efficiency test	>80%	Pass	Ok
7	3 passage growth test	>80%	Pass	Ok
8	Residue of exosomes	<3%	Pass	Ok

This document confirms that the above parameters have been tested and meet the quality control (QC) standard.

Signature: C.H.Deng **C.H.DENG** 2020.1.7
QC Department

Date: 2020.01.07

Electrophoresis & Brownian Motion
Video Analysis
Laser Scattering Microscopy

Operator (Report): Administrator
Video Operator: Administrator

Sample Parameters
Sample Name: A186-01-01
Comment: Sample Remarks0:
Sample Remarks1:
Sample Remarks2:
Electrolyte:
Temperature: 22.94 °C sensed
pH 7.0 entered
Conductivity: 0.00 µS/cm entered

Result (sizes in nm)

	Number	Concentration	Volume
Median (X50)	84.3	84.3	145.7
Span	43.0	43.0	89.3

Concentration: 1.7E+7 Particl es / mL
Dilution Factor: 400
Original Concentration: 7.0E+9 Particles / mL

Quality
Average Counted Particles per Frame: 190
Number of Traced Particles: 2359

Measurement Parameters
Cell S/N: NTA

Measurement Mode: Size Distribution 4 Cycles
11 Positions

Analysis Parameters
Max Area: 1000, Min Area: 5, Min Brightness: 20

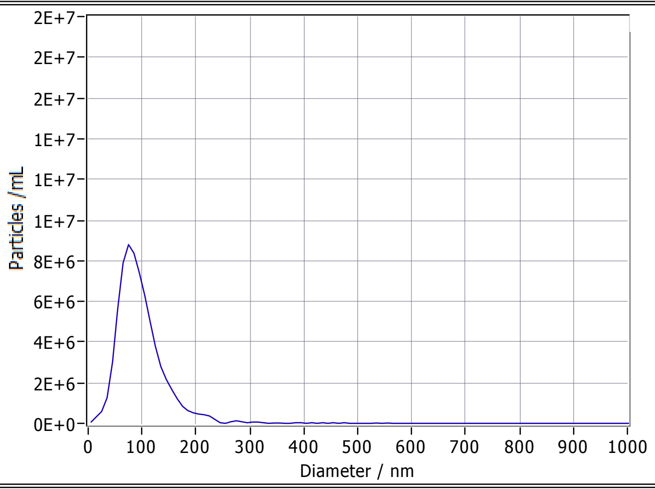

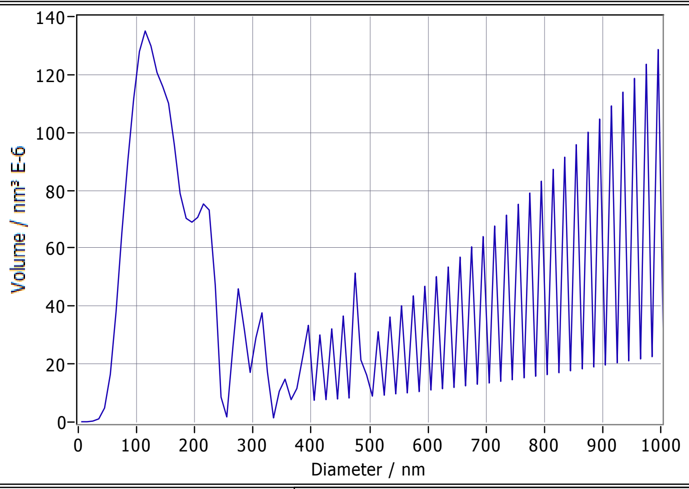

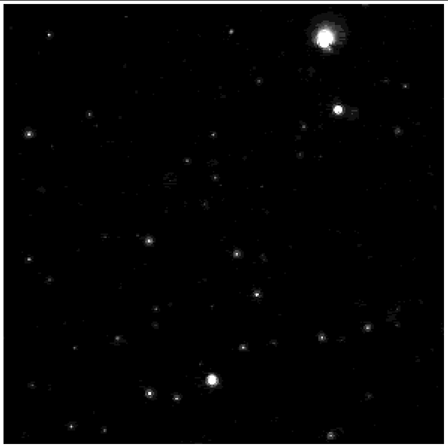

Peak Analysis (Concentration)

Diameter / nm	Particles/mL	FWHM / nm	Percentage
81.4	8.5E+6	69.8	97.4
303.7	5.5E+4	23.6	0.7
453.1	1.9E+4	109.0	0.1
356.8	1.7E+4	10.0	0.0
439.8	1.4E+4	0.0	0.0

X Values

	Number	Concentration	Volume
X10	48.2	48.2	82.9
X50	84.3	84.3	145.7
X90	143.5	143.5	287.1
Span	1.1	1.1	1.4
Mean	97.1	97.1	174.9
StdDev	43.0	43.0	89.3

Comment

(Signature)

Analyzed Video: E:\NTA Measurements\20200629_0002_A186-01-01_size.avi

ZetaVIEW S/N 18-373, Software ZetaView 8.04.02 SP2, Camera 0.771 µm/px

Experiment: 2020-06-29 11:26, Report: 2020-06-29 11:34

Electrophoresis & Brownian Motion
Video Analysis
Laser Scattering Microscopy

www.particle-metrix.de

Operator (Report): Administrator
Video Operator: Administrator

Sample Parameters
Sample Name: A186-01-02
Comment: Sample Remarks0:
Sample Remarks1:
Sample Remarks2:
Electrolyte:
Temperature: 23.39 °C sensed
pH 7.0 entered
Conductivity: 0.00 µS/cm entered

Result (sizes in nm)

	Number	Concentration	Volume
Median (X50)	82.4	82.4	143.3
Span	41.7	41.7	70.2

Concentration: 6.7E+7 Particles / mL
Dilution Factor: 400
Original Concentration: 2.7E+10 Particles / mL

Quality
Average Counted Particles per Frame: 181
Number of Traced Particles: 2217

Measurement Parameters
Cell S/N: NTA

Measurement Mode: Size Distribution 4 Cycles
11 Positions

Analysis Parameters
Max Area: 1000, Min Area: 5, Min Brightness: 20

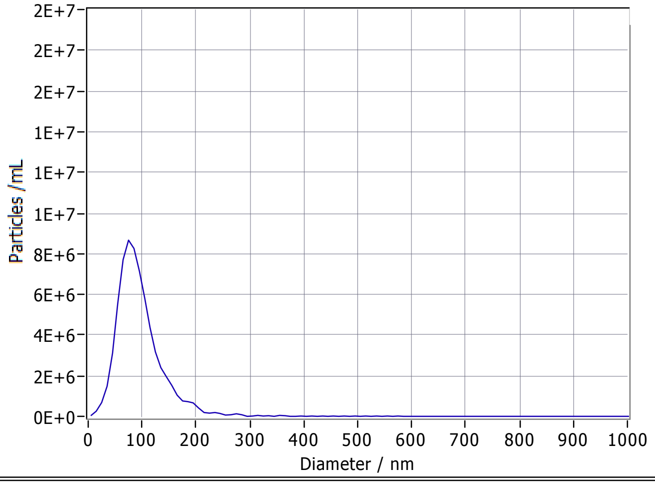

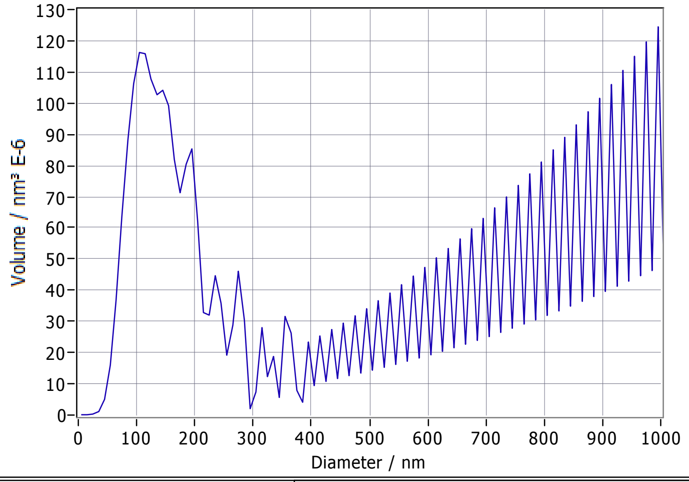

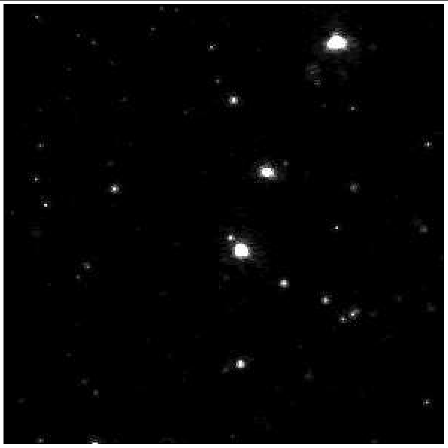

Peak Analysis (Concentration)

Diameter / nm	Particles/mL	FWHM / nm	Percentage
80.3	8.4E+6	65.4	97.5
362.2	3.4E+4	106.5	0.3
403.5	1.0E+4	10.0	0.0
561.4	8.5E+3	30.0	0.1
445.3	7.9E+3	11.6	0.2

X Values

	Number	Concentration	Volume
X10	47.3	47.3	80.5
X50	82.4	82.4	143.3
X90	142.9	142.9	260.6
Span	1.2	1.2	1.3
Mean	95.1	95.1	162.6
StdDev	41.7	41.7	70.2

Comment

(Signature)

Analyzed Video: E:\NTA Measurements\20200629_0003_A186-01-02_size.avi

ZetaVIEW S/N 18-373, Software ZetaView 8.04.02 SP2, Camera 0.771 µm/px

Experiment: 2020-06-29 11:36, Report: 2020-06-29 11:45

Electrophoresis & Brownian Motion
Video Analysis
Laser Scattering Microscopy

www.particle-metrix.de

Operator (Report): Administrator
Video Operator: Administrator

Sample Parameters
Sample Name: A186-02
Comment: Sample Remarks0:
Sample Remarks1:
Sample Remarks2:
Electrolyte:
Temperature: 23.82 °C sensed
pH 7.0 entered
Conductivity: 0.00 µS/cm entered

Result (sizes in nm)

	Number	Concentration	Volume
Median (X50)	79.1	79.1	132.8
Span	38.3	38.3	65.3

Concentration: 9.5E+7 Particles / mL
Dilution Factor: 400
Original Concentration: 3.8E+10 Particles / mL

Quality
Average Counted Particles per Frame: 258
Number of Traced Particles: 2803

Measurement Parameters
Cell S/N: NTA

Measurement Mode: Size Distribution 4 Cycles
11 Positions

Analysis Parameters
Max Area: 1000, Min Area: 5, Min Brightness: 20

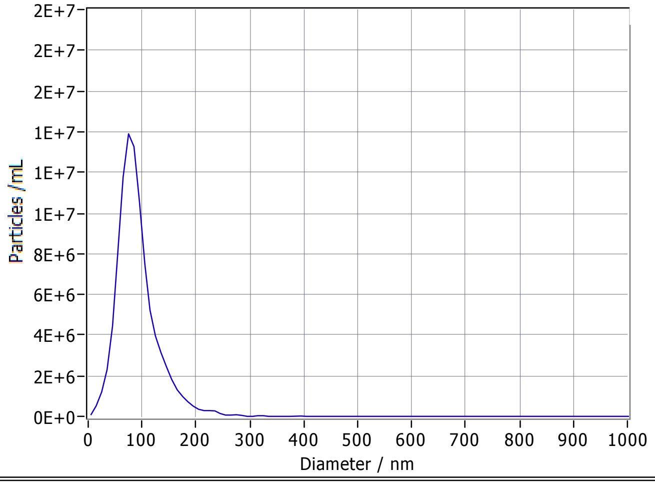

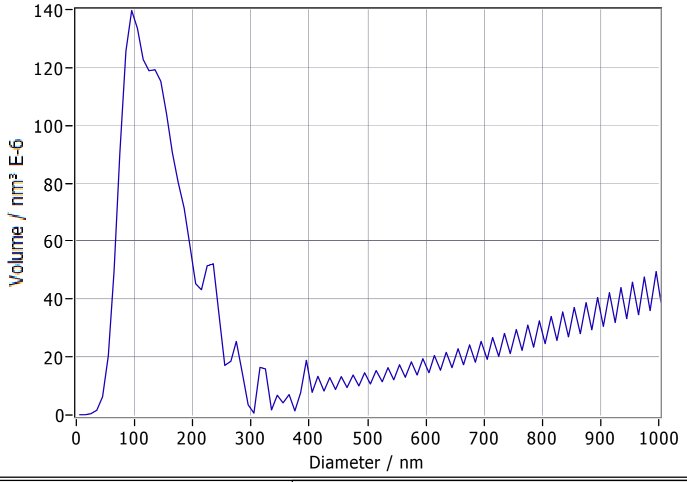

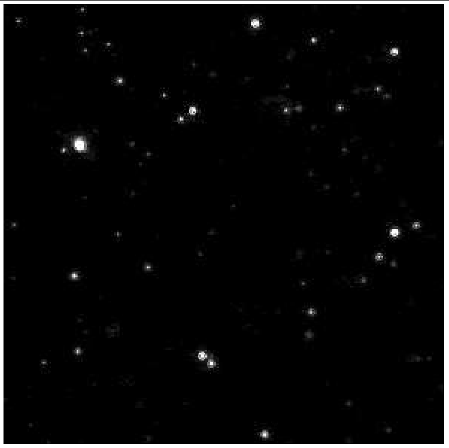

Peak Analysis (Concentration)

Diameter / nm	Particles/mL	FWHM / nm	Percentage
78.6	1.4E+7	55.2	99.0
315.5	3.4E+4	10.0	0.0
403.8	9.0E+3	15.6	0.1
445.3	6.5E+3	14.8	0.1
354.8	6.1E+3	20.0	0.0

X Values

	Number	Concentration	Volume
X10	46.3	46.3	75.9
X50	79.1	79.1	132.8
X90	134.7	134.7	231.8
Span	1.1	1.1	1.2
Mean	90.8	90.8	150.6
StdDev	38.3	38.3	65.3

Comment

(Signature)

Analyzed Video: E:\NTA Measurements\20200629_0004_A186-02_size.avi

ZetaVIEW S/N 18-373, Software ZetaView 8.04.02 SP2, Camera 0.771 µm/px

Experiment: 2020-06-29 11:46, Report: 2020-06-29 11:55

Electrophoresis & Brownian Motion
Video Analysis
Laser Scattering Microscopy

www.particle-metrix.de

Operator (Report): Administrator
Video Operator: Administrator

Sample Parameters
Sample Name: A186-03
Comment: Sample Remarks0:
Sample Remarks1:
Sample Remarks2:
Electrolyte:
Temperature: 24.29 °C sensed
pH 7.0 entered
Conductivity: 0.00 µS/cm entered

Measurement Parameters
Cell S/N: NTA

Measurement Mode: Size Distribution 4 Cycles
11 Positions

Result (sizes in nm)

	Number	Concentration	Volume
Median (X50)	86.7	86.7	143.8
Span	42.0	42.0	74.3

Concentration: 6.5E+7 Particles / mL
Dilution Factor: 800
Original Concentration: 5.2E+10 Particles / mL

Quality
Average Counted Particles per Frame: 177
Number of Traced Particles: 2505

Analysis Parameters
Max Area: 1000, Min Area: 5, Min Brightness: 20

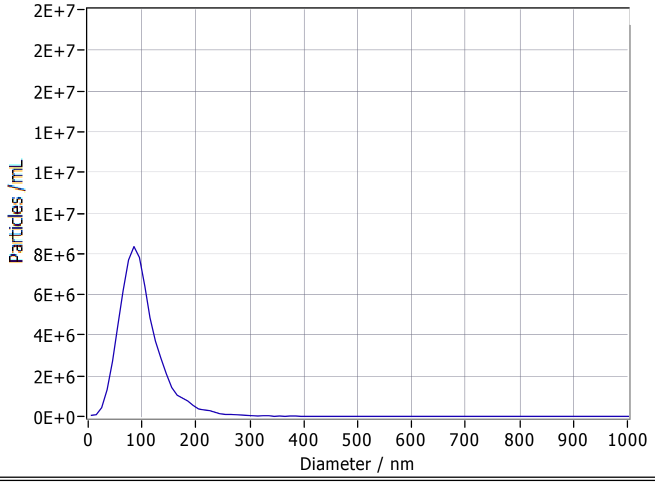

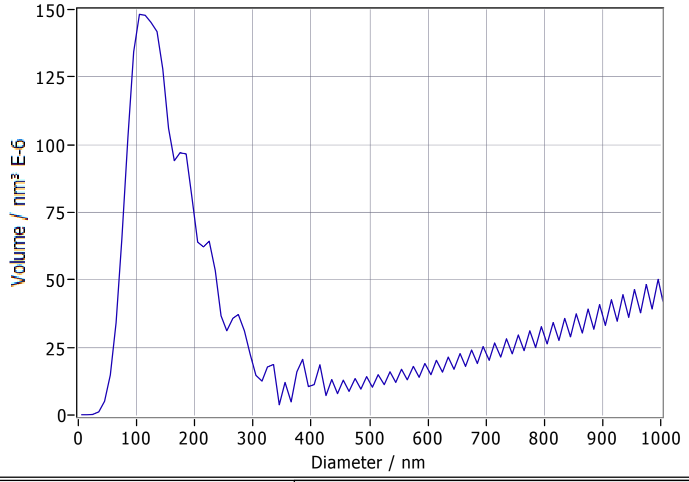

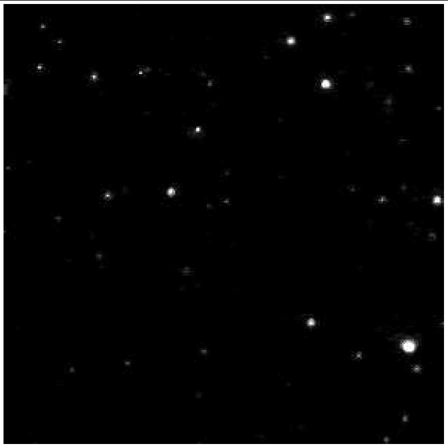

Peak Analysis (Concentration)

Diameter / nm	Particles/mL	FWHM / nm	Percentage
86.2	8.3E+6	67.0	98.5
303.4	2.9E+4	30.7	0.3
409.2	1.0E+4	65.5	0.1
359.3	9.8E+3	30.0	0.1
463.8	4.6E+3	30.0	0.0

X Values

	Number	Concentration	Volume
X10	49.4	49.4	83.6
X50	86.7	86.7	143.8
X90	144.8	144.8	265.8
Span	1.1	1.1	1.3
Mean	98.9	98.9	166.6
StdDev	42.0	42.0	74.3

Comment

(Signature)

Analyzed Video: E:\NTA Measurements\20200629_0005_A186-03_size.avi

ZetaVIEW S/N 18-373, Software ZetaView 8.04.02 SP2, Camera 0.771 µm/px

Experiment: 2020-06-29 11:58, Report: 2020-06-29 12:07
